# Exploiting Friedel pairs to interpret scanning 3DXRD data from complex geological materials

**DOI:** 10.1107/S1600576724009634

**Published:** 2024-11-08

**Authors:** Jean-Baptiste Jacob, Jonathan Wright, Benoît Cordonnier, François Renard

**Affiliations:** a The Njord Centre, Departments of Geosciences and Physics University of Oslo Norway; b The European Synchrotron Radiation Facility (ESRF) Grenoble France; c ISTerre, Univ. Grenoble Alpes, Grenoble INP, Univ. Savoie Mont Blanc, CNRS, IRD, Univ. Gustave Eiffel, Grenoble, France; Ecole National Supérieure des Mines, Saint-Etienne, France

**Keywords:** synchrotron X-ray diffraction, 3DXRD, Friedel pairs, geological materials

## Abstract

The present study introduces a processing strategy for synchrotron scanning 3D X-ray diffraction (s3DXRD) data, aimed at addressing the challenges posed by large, highly deformed, polyphase materials such as crystalline rocks. Leveraging symmetric Bragg reflections known as Friedel pairs, our method enables diffraction events to be precisely located within the sample volume. This method allows for fitting the phase, crystal structure and unit-cell parameters at the intra-grain scale on a voxel grid. The processing workflow incorporates several new modules, designed to (i) efficiently match Friedel pairs in large s3DXRD datasets containing up to 10^8^ diffraction peaks; (ii) assign phases to each pixel or voxel, resolving potential ambiguities arising from overlap in scattering angles between different crystallographic phases; and (iii) fit the crystal orientation and unit cell locally on a point-by-point basis. We demonstrate the effectiveness of our technique on fractured granite samples, highlighting the ability of the method to characterize complex geological materials and show their internal structure and mineral composition. Additionally, we include the characterization of a metal gasket made of a commercial aluminium alloy, which surrounded the granite sample during experiments. The results show the effectiveness of the technique in recovering information about the internal texture and residual strain of materials that have undergone high levels of plastic deformation.

## Abstract 2

A new processing technique for synchrotron scanning 3D X-ray diffraction data is introduced, utilizing symmetric Bragg reflections *hkl* and *h**k**l*, known as Friedel pairs. This technique is designed to tackle the difficulties associated with large, highly deformed, polyphase materials, especially geological samples.

## 1. Introduction

Three-dimensional X-ray diffraction (3DXRD), or high-energy diffraction microscopy (HEDM), are synchrotron diffraction techniques based on the rotation method with hard monochromatic X-rays (10–100 keV). They allow the non-destructive investigation of the internal structure of polycrystalline materials on a per-grain basis, with a penetration depth of up to a few millimetres (Bernier *et al.*, 2011 ▸; Bernier *et al.*, 2020 ▸; Poulsen *et al.*, 2004 ▸; Poulsen, 2012 ▸). The technique relies on the measurement of a series of spotty diffraction images on a 2D detector during the rotation of the sample relative to the incident X-ray beam. Each diffraction spot comes from the crystal lattice planes of each grain when it passes through the Bragg diffraction condition. Provided spot overlap is not excessive, the diffraction spots can be segmented into discrete diffraction peaks. These peaks are then assigned to grains, a process called ‘indexing’. This process is a search–match of known crystalline phases to determine the unit cell, followed by an orientation search (Bernier *et al.*, 2011 ▸; Ludwig *et al.*, 2009 ▸; Schmidt, 2014 ▸; Wright, 2024 ▸). The indexed data allow per-grain estimation of the position, orientation, shape and unit cell of hundreds to thousands of grains simultaneously. If a reference unit cell is available, then elastic strain and stress can be derived from the unit-cell parameters.

Since its first introduction about two decades ago (Poulsen *et al.*, 2004 ▸), the method has been refined and deployed in various synchrotron radiation facilities globally. It has diversified into various techniques, using either ‘far-field’ or ‘near-field’ acquisition modes and different beam geometries. Near-field setups use a detector pixel size much smaller than the X-ray beam size (*e.g.* a pixel size of a few µm, sample-to-detector distances from ∼2 to ∼20 mm). This geometry optimizes the information on grain shapes and position and allows complete tomographic reconstruction of the grains in the sampled volume (Ludwig *et al.*, 2009 ▸; Reischig *et al.*, 2013 ▸). In contrast, far-field setups use larger pixel detectors that are further back from the sample (pixel size >20 µm, sample-to-detector distance from ∼50 to 2000 mm). The far-field data are dominated by angular dispersion and optimize recovery of the diffraction pattern to determine grain orientation and strain, but reduce the precision on grain position and shape in the results (Poulsen, 2012 ▸). Different beam geometries can be used, ranging from a wide 2D ‘box beam’ covering a 3D volume in the sample, through a ‘line’ focus that illuminates a 2D slice across the sample, to a point-focused beam illuminating only a thin pencil line across the sample. The 3DXRD technique and its derivatives have become popular tools for the materials science community and have been successfully applied to a wide range of topics, including recrystallization, annealing, plastic deformation, fatigue crack growth in metals and mechanical loading of granular materials (Hurley *et al.*, 2018 ▸; Naragani *et al.*, 2017 ▸; Oddershede *et al.*, 2012 ▸; Thakur *et al.*, 2023 ▸).

However, 3DXRD still faces major challenges in investigations of complex samples, such as highly deformed and/or multi-phase materials. This is especially the case for geological materials. First, they commonly feature hierarchical structures, with a large range of grain sizes that can span from sub-micrometre-size crystallites to millimetre- or centimetre-size crystals within the same sample (*e.g.* for detrital sedimentary rocks containing large clasts cemented in a thin matrix). This complexity results in diffraction patterns that feature a mix of discrete spots and more continuous rings, similar to powder diffraction. Although 3DXRD analysis only works with discrete spots, other techniques such as diffraction tomography (Artioli *et al.*, 2010 ▸) and texture tomography (Frewein *et al.*, 2024 ▸; Mürer *et al.*, 2021 ▸; Zhao *et al.*, 2024 ▸) can be applied to extract information on crystal phase and orientation from the continuous rings.

Second, geological materials generally display various degrees of brittle and/or ductile deformation, as a consequence of their tectonic history. This results in the presence of cracked crystals, twins, low-angle grain boundaries and large internal misorientation gradients within single grains. All these features complicate the segmentation of the diffraction spots on the detector. Fragmented grains with low misorientation form clusters of closely grouped spots, which are difficult to separate. Twins have systematically overlapping diffraction peaks. Plastic strain results in more diffuse spots, covering a large azimuthal range and resulting in peak overlaps. After segmentation, indexing of these peaks is also challenging. The large spread of orientations in plastically deformed grains implies that the orientation distribution function of each grain covers a relatively wide domain in the orientation space, with a common overlap between the orientation distribution functions of spatially disconnected grains. Thus, grain indexing strategies based on a search–match process in reciprocal space can yield misleading results.

Then, problems arise from the fact that there is no canonical definition of what a grain is. Grain boundaries can be defined on the basis of a misorientation threshold between two contiguous domains, but the threshold angle is an arbitrary value. In materials that have crystallized statically, *e.g.* during an annealing process, this is usually not a problem, because the grains are delimited by sharp high-angle discontinuities and do not show a large internal misorientation (Passchier & Trouw, 2005 ▸). In materials with a large strain, or which have undergone dynamic recrystallization, there is significant spatial heterogeneity in terms of grain size, grain boundary misorientation and internal grain misorientation (Passchier & Trouw, 2005 ▸), which implies that there is no best threshold to be chosen. This is a fundamental problem for indexing because grain segmentation (‘grain mapping’) and fitting of orientation and unit cell are done simultaneously over the whole sample, using a single set of threshold criteria to decide which peaks are assigned to which grain. Therefore, a given set of indexing parameters cannot capture all the microstructural features of a rock at the same time. For example, a given set of indexing parameters may be suitable for identifying large grains with large plastic strain and internal misorientation but may be ineffective in separating a cluster of small grains delimited by low-angle grain boundaries.

Rocks typically consist of complex mineral assemblages, many of which have low symmetry (monoclinic or triclinic) and/or form complex solid solutions with spatial composition zoning. Low symmetry results in a large number of independent scattering angles (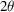) for each phase, related to the different {*hkl*} families. Compositional zoning also adds variability to the unit cell. Finally, large samples, at least a few millimetres wide, are usually required to obtain a representative rock volume (Thakur *et al.*, 2023 ▸), implying that the position of the diffraction peaks in the detector is significantly affected by the position of the grains within the sample. Altogether, these effects result in complex datasets, in which diffraction spots are not positioned on the Debye–Scherrer rings familiar from powder diffraction, as usually observed in far-field 3DXRD. Assigning diffraction peaks to a phase, a prerequisite step for indexing, is therefore not trivial, and often cannot be performed using simple thresholding around a set of pre-computed 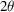 angles for each phase.

These issues have greatly hampered the application of 3DXRD to geoscience problems, despite its huge potential for *in situ* mineral characterization during experiments, non-destructive investigation of rare and valuable samples, or investigation of strain and stress related to rock deformation. Studies undertaken so far on geological materials have been limited to single-phase samples such as rock salt (Alshibli *et al.*, 2021 ▸; Borthwick *et al.*, 2012 ▸) or artificial analogs, for example replacing natural quartz grains with hydrothermally grown artificial quartz (Hurley *et al.*, 2016 ▸; Hurley *et al.*, 2018 ▸; Thakur *et al.*, 2023 ▸).

The different variations of the 3DXRD/HEDM technique each have their own advantages and drawbacks, which make them variably suitable for investigating geological materials. Near-field techniques, such as near-field high-energy X-ray diffraction microscopy (nf-HEDM) and diffraction contrast tomography (DCT), require full-field illumination of the sample, with either a line beam or a box beam (Ludwig *et al.*, 2009 ▸; Bernier *et al.*, 2020 ▸). This approach has the advantage of relatively fast acquisition, and recent developments have enabled the retrieval of intra-grain orientation and strain with reasonable precision (Shen *et al.*, 2020 ▸; Reischig & Ludwig, 2020 ▸). These setups are well suited for samples up to a few millimetres in size with a limited number of grains. Typically, these techniques may be the perfect fit for investigating micro-inclusions in isolated minerals such as zircon or diamond, with direct application for instance in mantle petrology (Walter *et al.*, 2011 ▸) or for studying early Earth processes in Hadean zircon (Harrison *et al.*, 2017 ▸). However, they are unsuitable for large, highly deformed samples, as spot overlaps are almost inevitable in such cases, which can impede accurate reconstruction using DCT/nf-HEDM algorithms (Shen *et al.*, 2020 ▸; Reischig & Ludwig, 2020 ▸).

For such large and/or deformed samples, the pencil beam scanning technique (Hayashi *et al.*, 2015 ▸), known as scanning 3DXRD (s3DXRD), seems the most promising method. This technique employs a pencil beam to illuminate thin sub-volumes of the sample one at a time, thereby reducing diffraction spot overlap on the detector. However, the acquisition time is considerably extended compared with that for regular 3DXRD or DCT, as the sample must undergo multiple translations and rotations to cover a full slice. This scanning technique facilitates the mapping of intra-grain orientation and strain fields, through either a point-by-point fitting procedure (Hayashi *et al.*, 2017 ▸) or more advanced regression methods exploiting tomographic principles (Henningsson *et al.*, 2020 ▸; Henningsson & Hendriks, 2021 ▸). Point-by-point fitting also has the advantage of being agnostic to the notion of grain, because it is performed on a fixed grid of pixels/voxels that does not depend on the definition of grain boundaries.

However, several challenges remain before the technique can be applied to geological samples. The representative volume element required for rock samples is at least a few millimetres and this gives significant shifts in 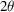 peak positions due to the sample thickness. Algorithms exist to address this problem for powder data with continuous diffraction rings (Liang *et al.*, 2022 ▸; Vamvakeros *et al.*, 2020 ▸), but for spotty data the usual far-field indexing approaches assume that 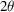 angles are known. Moreover, the challenges posed by 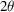 overlaps between different phases remain unresolved. Solutions using conical or spiral slits have been proposed to physically filter the range of scattering angles captured by the detector (Lienert *et al.*, 2000 ▸; Hayashi *et al.*, 2019 ▸; Hayashi *et al.*, 2023 ▸), which reduces spot overlap and allows the selection of peaks arising from a chosen phase or a sub-region in the sample. However, this approach is limited to certain crystal symmetries and does not allow simultaneous data acquisition from many phases.

The present study proposes a new experimental and processing strategy for s3DXRD data, specifically designed to tackle the multiple challenges raised by large, highly deformed, polyphasic materials, particularly rocks. A key element of this procedure is the use of symmetric Bragg reflections *hkl*, *h**k**l*, also known as Friedel pairs, which are measured using inverse beam geometry (180° rotation). These pairs determine the direction of scattering vectors and the location from which they arise in the sample. This approach combines the ray tracing of Friedel pairs from a DCT experiment (Ludwig *et al.*, 2009 ▸) with the tomographic information from s3DXRD scanning (Bonnin *et al.*, 2014 ▸; Hayashi *et al.*, 2017 ▸) to give the maximal spatial and angular information from experiments. Friedel pairs are then mapped on a 2D pixel grid, allowing phases to be labeled in each pixel, with subsequent local fitting of local crystal lattice orientations and unit cells. This procedure can be extended to a 3D voxel grid by stacking multiple 2D slices.

The article is structured as follows. The next section offers a comprehensive description of the method, detailing each step involved in generating a processed grain map from a set of diffraction peaks segmented from 2D diffraction frames. The following section demonstrates the application of the data processing pipeline on two distinct datasets obtained from large samples of granite (*ca* 5 mm wide) at different scanning resolutions. Finally, the last section discusses the advantages, existing limitations and potential future improvements of this technique.

## 2. Method

### 2.1. Prerequisites and overview of the workflow

The method is designed to process s3DXRD data. This implies that data collection has been done using a pencil beam scanning procedure, with a series of constant-speed rotations along the vertical axis of the sample, and translating the sample in a direction orthogonal to the plane formed by the beam and the rotation axis after each rotation. A full 360° rotation is needed to recover symmetrical *hkl* and *h**k**l* reflections for each crystal plane. In the following, notations defined by Poulsen & Vaughan (2019 ▸) for the 3DXRD geometry setup are employed. Coordinates 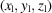 correspond to the fixed Cartesian laboratory reference frame, and coordinates 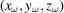 correspond to the sample reference frame, obtained by successively applying a translation 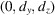 and a rotation of angle ω along the *z* axis. By convention, the incident X-ray beam is parallel to 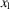, and the rotation axis *z* is taken as close as possible from the vertical axis 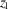, although a small misalignment (wedge angle) inevitably occurs. During scanning, translations in the 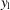 direction are repeated from positions 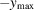 to 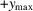, with an increment 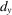 greater than or equal to the beam width. If the acquisition is performed on a 3D volume, the scanning procedure is repeated from *z* position 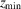 to 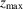 with an increment 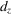 between each slice.

The data processing pipeline starts from segmented diffraction peaks obtained after pre-processing the raw diffraction images. Pre-processing here includes accurate calibration of the detector geometry (detector distance, center, tilt and wedge) using a calibration material (*e.g.* NIST CeO_2_ powder), subtraction of background noise, if needed, from raw diffraction frames, segmentation of diffraction spots into discrete diffraction peaks, and correction of peak coordinates using detector geometry parameters previously fitted with the calibration data. Peaks are characterized by the center-of-mass coordinates of connected components – ‘spots’ – in 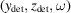 space, where 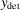 and 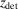 are spatially corrected detector coordinates and ω is the rotation along the *z* axis. The peak intensity *I* corresponds to the integrated intensity of the corresponding 3D spot. The method also works on 2D spots, which are not integrated along ω, but the pros and cons of working on 2D versus 3D peaks will not be discussed here. Finally, the method extensively relies on *ImageD11* Python tools (Wright, 2024 ▸), especially regarding manipulation and storage of diffraction peak data and local fitting of lattice orientation and unit cell.

**Figure 1:**
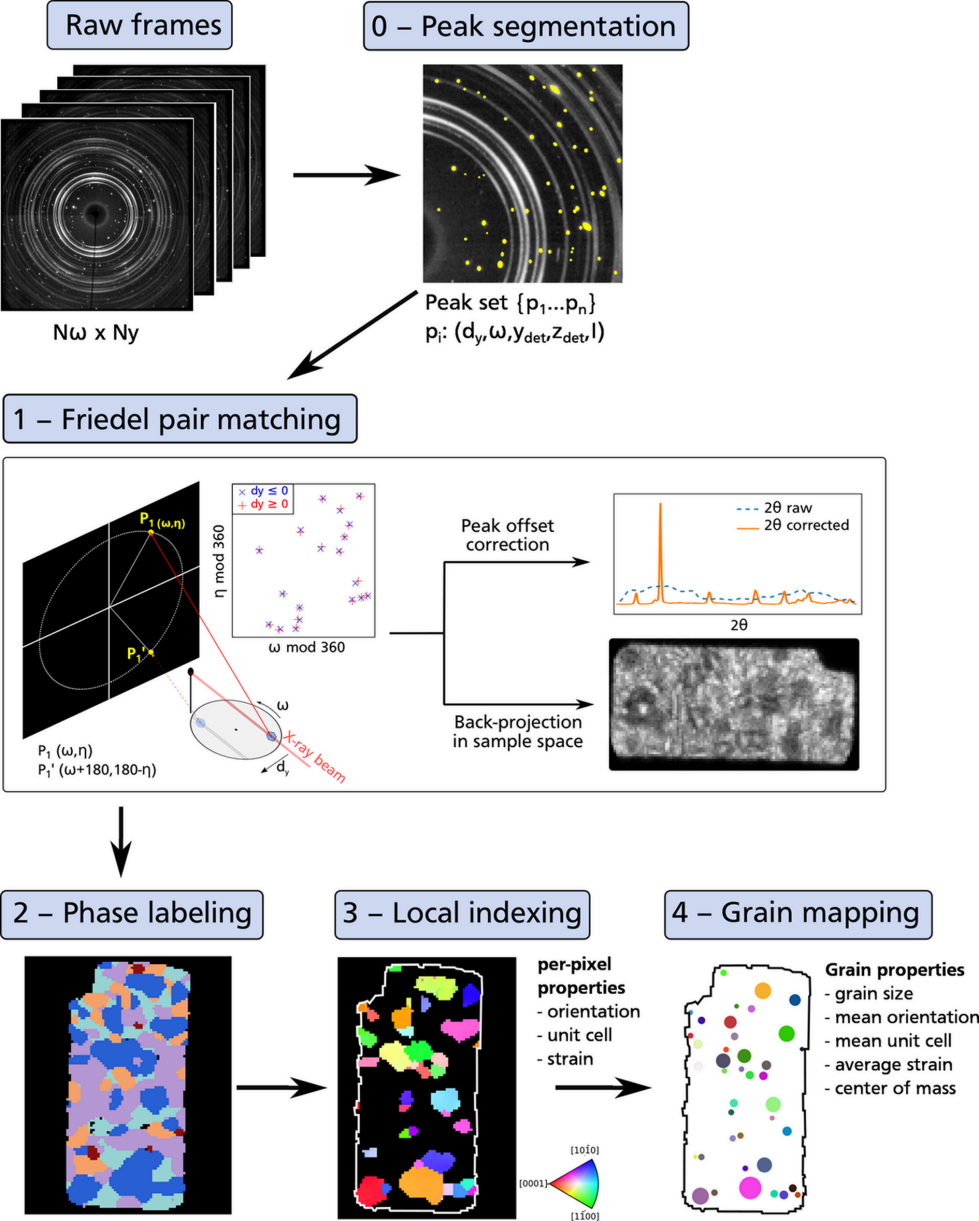
Schematic workflow for processing s3DXRD data using the Friedel pairs method. The four steps to obtain a processed grain map from a set of segmented diffraction peaks are detailed in Section 2.

The processing workflow consists of four different steps in addition to pre-processing, which are summarized in Fig. 1 ▸: (i) Friedel pair identification; (ii) point-by-point phase labeling; (iii) point-by-point fitting of crystal orientation and unit cell; (iv) grain mapping. A more detailed flowchart is available in the supporting information. Identifying the different phases in the sample involves azimuthal integration of the diffraction data to generate a powder-like diffraction pattern, which can then be processed by Rietveld or Le Bail analysis to identify phases and refine crystal structures (Rietveld, 1969 ▸; Le Bail *et al.*, 1988 ▸). Strain and stress analysis from point-by-point fitted pixel/grain maps is also straightforward from the map obtained by local indexing (Fig. 1 ▸) but will not be discussed here.

### 2.2. Friedel pairs

Friedel pairs are symmetric Bragg reflections arising from the (*hkl*) and (*h**k**l*) planes of a grain, which occur 180° apart during a full 360° rotation of the sample perpendicularly to the beam. Their symmetric properties allow the separation of the component of a scattered wavevector related to the lattice state (spacing and orientation) – the ‘true’ scattered wavevector – from the component related to the offset from the rotation center, considerably simplifying data analysis. Indexing strategies based on Friedel pairs have been implemented for a long time for near-field setups, in particular for DCT (Ludwig *et al.*, 2009 ▸; Reischig *et al.*, 2013 ▸). They have also been proposed for 3DXRD in a box beam setting (Moscicki *et al.*, 2009 ▸) but, to our knowledge, they have so far not been used for s3DXRD.

#### 2.2.1. Friedel pair geometry

**Figure 2:**
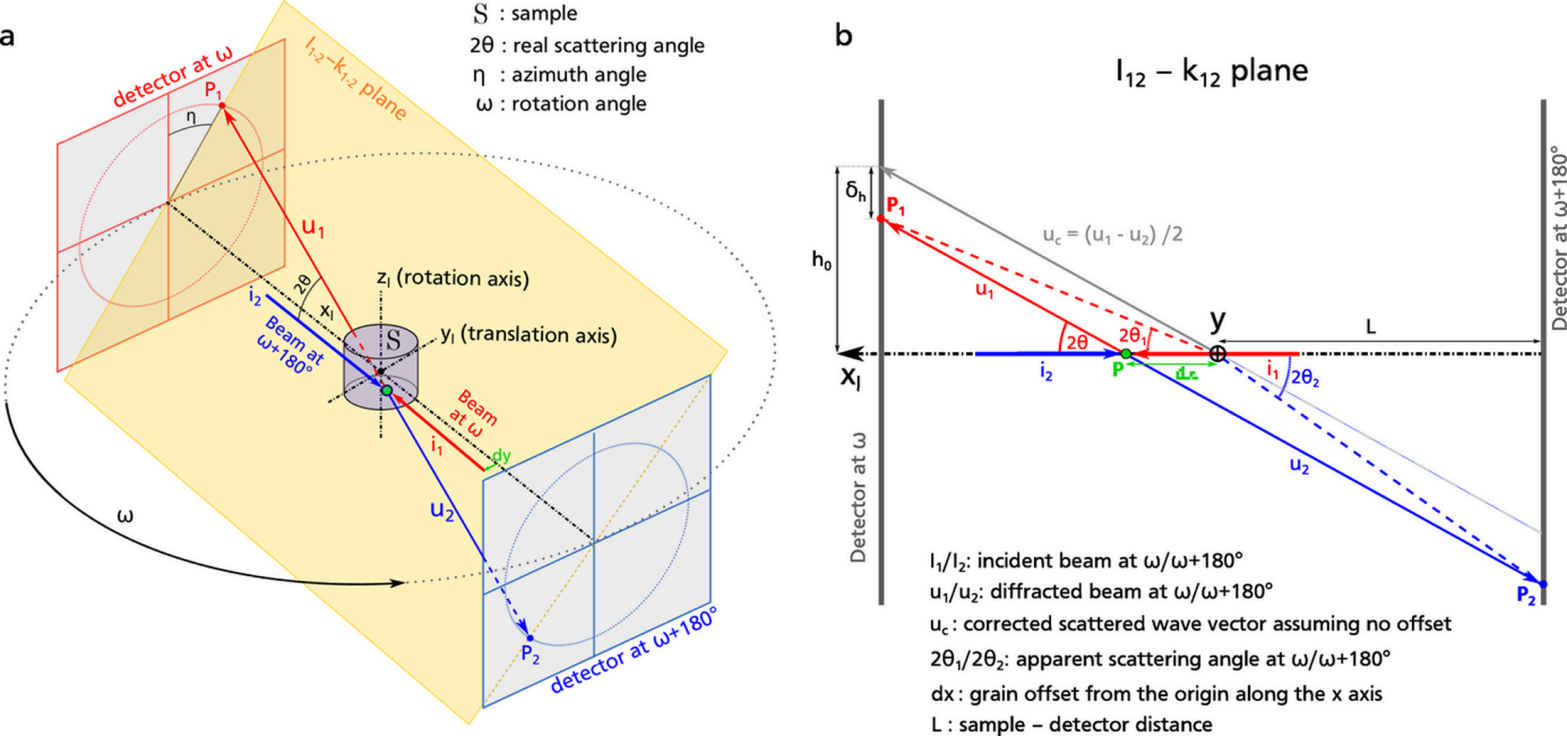
Friedel pair geometry for a s3DXRD acquisition in a reference frame fixed relative to the sample. (*a*) Sketch of the setup, with two detector positions at angles ω and ω + 180°, and an arbitrary translation 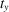 of the sample relative to the rotation center. A single *hkl* reflection arising from the green spot in the sample at rotation angle ω is shown, together with its paired reflection *h**k**l* occurring at ω + 180°. The two incident beams 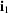 and 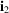 are thin pencil beams of negligible dimension in *y* and *z*, and are coincident. The two diffracted beams 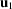 and 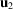 are also coincident and intersect the detector at 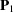 and 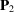, respectively. (*b*) View of the plane containing 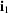, 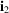, 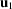 and 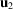. 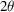 is the real scattering angle between 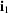 and 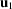 (or 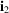 and 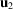), and η is the azimuth angle on the detector. 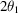 and 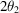 are apparent scattering angles, computed assuming that the diffracting point in the sample has no offset along the *x* axis, *i.e.* it is at distance *L* from the detector. 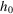 is the radial distance from the detector center to the spot, which would be observed both for 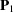 and 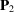 if there was no offset along the *x* axis. 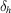 is the offset from this position resulting from the offset 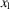 of the diffracting source along the *x* axis.

As pointed out by Ludwig *et al.* (2009 ▸), the benefits of Friedel pairs are better visualized in a reference frame where the sample is fixed and the detector and the beam are rotated around it. In this setup, it becomes clear that two symmetrical reflections *hkl* and *h**k**l* arising from the same origin in the sample form two parallel vectors 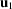 and 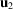 pointing in opposite directions [Fig. 2 ▸(*a*)]. Thus, the orientation of the true (offset-corrected) scattered wavevector is given by 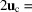

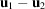
, independently of the origin in the sample. This allows us to retrieve the accurate diffraction angles 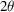 and the azimuth angles η from peak coordinates on the detector, without *a priori* knowledge of where the diffracted X-rays arise from in the sample. Moreover, the norm of 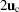 is approximately twice the norm of 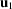 and 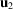 [Fig. 2 ▸(*a*)], effectively doubling the sample–detector distance and increasing angular resolution.

With regular (box beam) 3DXRD, a unique Friedel pair 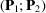 is insufficient to fit the position of the diffracting region 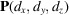 in the sample. Indeed, if 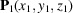 and 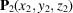 are the intersections of the two diffracted rays with the detector at position 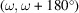, the set of possible solutions for 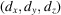 corresponds to the intersection between the line 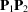 and the sub-volume of the sample *S* illuminated by the beam [Fig. 2 ▸(*a*)], which corresponds to a linear segment 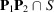, with
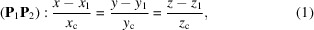
where 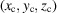 are the coordinates of the offset-corrected scattered wavevector 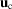 parallel to 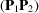. This system is under-determined with one degree of freedom, meaning that any combination of two coordinates can be written as a function of the third one, but all three cannot be constrained at the same time. To address this limitation, it is possible to identify the four peaks, constituting two Friedel pairs, arising from a single set of (*hkl*), (*h**h**k*) crystal planes during a full 360° rotation. Grain center-of-mass positions are then fitted using a least-squares approach, considering all experimentally found Friedel pairs for each grain (Ludwig *et al.*, 2009 ▸; Moscicki *et al.*, 2009 ▸).

With s3DXRD data, the use of a thin pencil beam simplifies the problem and eliminates the need to match the four peaks for each (*hkl*) plane. Indeed, the beam size in the *y* and *z* directions is typically smaller than the pixel size of the detector, reducing the illuminated volume to a thin line in the sample. The diffracting region then corresponds to the intersection point between this line segment and 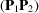, which can be fully constrained from the peak positions on the detector.

In other words, for a given rotation ω of the sample, to illuminate a specific point 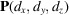 in the laboratory reference frame, the sample must be translated by a precise distance 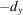 and 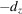 along the laboratory *y* and *z* axes, respectively, to position **P** in the beam path [Fig. 2 ▸(*a*)]. Therefore, 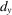 and 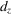 are constrained by the sample translation needed to place **P** within the incident X-ray path, leaving only the *x* coordinate 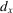 along the beam as unknown. The lack of offset in the *y* and *z* directions also implies that the offset affects only the 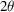 angle, while the azimuth angle η remains unmodified. This is better visualized in the plane defined by the incident beam and the two scattered wavevectors 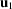 and 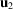 [Fig. 2 ▸(*b*)]. 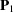 and 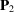 are, respectively, the intersection of 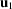 and 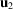 with the detector, *L* is the sample–detector distance, 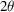 is the true scattering angle, and 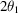 and 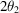 are, respectively, the apparent scattering angles for 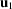 and 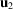, modified by the *x* offset 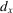. Using trigonometry relationships, it can be demonstrated that
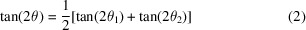
and 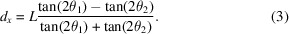
The diffraction source position in the sample reference frame is then obtained by applying successively a translation and a rotation to the point 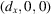 in the laboratory frame: 
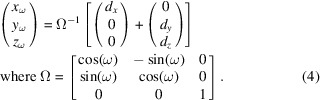
Thus, assuming that a Friedel pair has been correctly identified, the true orientation of the scattering vector and the position of the diffracting region in the sample are derived from (2), (3) and (4). However, these relationships depend on the assumption that the diffracting source can be approximated as a single point, which is typically not accurate. While the beam width constrains the *y* and *z* coordinates of the diffracting volume, only the average *x* position is determined by (3) and (4). The spread of the source along the *x* direction remains unknown.

Peak source coordinates 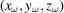 in the sample frame are then mapped onto a grid, with the voxel size set by the width of the incident beam. This grid can be used to obtain a reconstruction of the scanned volume, by computing the sum of diffracting intensity within each voxel. The result is similar to diffraction tomography maps obtained by applying the inverse Radon transform on the global sinogram of diffraction intensity (Artioli *et al.*, 2010 ▸).

#### 2.2.2. Finding Friedel pairs

To exploit the properties of Friedel pairs, these pairs need to be identified first. The pair-matching algorithm employed for DCT can reliably identify pairs in datasets of 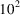
–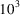 grains, typically yielding 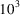
–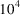 pairs of spots (Ludwig *et al.*, 2009 ▸). However, the number of peaks in a s3DXRD experiment is considerably larger and typically increases with higher scanning resolution (smaller beam size and thinner angular integration step size), larger sample size or a greater number of grains in the sample. For the geological samples investigated in this study (5 mm diameter), the number of peaks in a single 360° rotation scan typically ranges between 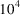 and 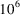, depending on the scanning resolution, and the total peak count reaches up to 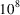 peaks for the highest-resolution dataset. Pair identification in such large datasets requires a computationally efficient algorithm to complete the task in a reasonable time.

**Figure 3:**
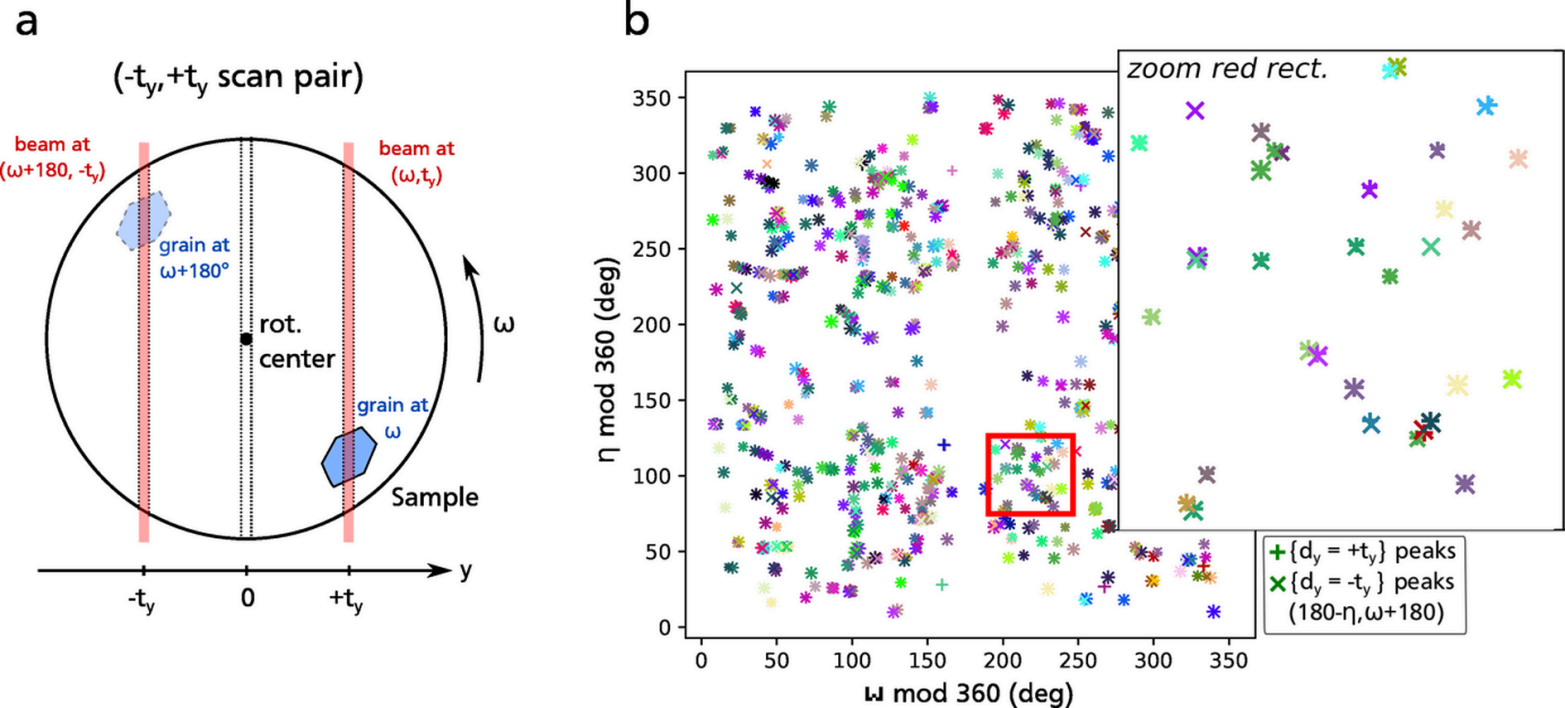
Friedel pair identification in a s3DXRD dataset. (*a*) Possible pairs are found in symmetric scans acquired at opposite *y* translation 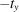 and 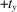 from the rotation center 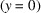. (*b*) 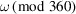 versus 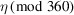 for a subset of paired peaks from symmetric scans (×: 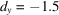; +: 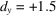) in sample WGSI3, after transforming 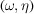 coordinates of the subset 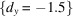 to make paired peaks overlap. Peaks are colored by Friedel pair index, a unique identifier for each pair in the dataset. Thus, two peaks forming a correct Friedel pair appear as overlapping ‘×’ and ‘+’ symbols of the same color. Spot size depends on log(intensity). Overall, there is a good match of Friedel pairs in ω and η. Isolated data points belong to unreliable pairs, where two peaks with very different 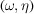 coordinates were associated together.

The strategy employed exploits the symmetry of the s3DXRD data acquired by doing complete 360° rotations along the *z* axis. Assuming that a null translation (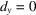) corresponds to the alignment of the pencil beam with the rotation center, two scans acquired with translations 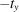 and 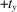 of the sample along the *y* axis [Fig. 3 ▸(*a*)] should contain the same information, except that 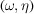 coordinates of the diffraction peaks acquired at 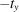 are flipped compared with those acquired at 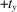. More precisely, applying the transformation 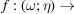

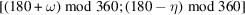
 to the subset of peaks 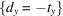 leads to a very good overlap with peaks in the subset 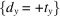 [Fig. 3 ▸(*b*)], which provides a good starting guess for matching Friedel pairs. Two peaks forming a pair should also display similar intensity *I* and scattering angle 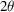. Thus, the pair-matching problem can be framed as a nearest-neighbor search in a 4D space formed using 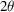, η, ω and *I*. Because these four variables represent different quantities, re-scaling is needed to form a consistent 4D Euclidian space in which a distance matrix can be computed. For intensity, logarithmic scaling is used because the peak intensity spans over several orders of magnitude and significant intensity variations are better represented on a logarithmic scale than on a linear scale. Logarithmic re-scaling is also needed for 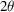, because variations in apparent 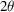 related to the geometrical offset 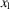 become larger with increasing scattering angle. In contrast, the difference in 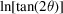 only depends on the detector distance *L* and geometrical offset 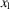.

The nearest-neighbor search is done in a computationally efficient way using *k*-dimensional tree structures (KD-trees), implemented in *Scipy* (Virtanen *et al.*, 2020 ▸). KD-trees are space-partitioning data structures built by recursive binary splitting of the *k*-dimensional space using axis-aligned hyperplanes, which allow efficient identification of nearest neighbors by quickly eliminating large portions of the search space (Maneewongvatana & Mount, 1999 ▸). Two KD-trees are computed, for two symmetric subsets of peaks 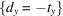 and 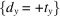. Then, a sparse distance matrix between the two subsets is computed, writing values in the matrix only when the distance between two peaks lies below a specified threshold. This sparse matrix is screened to retain only the smallest value in each row and column, ensuring each pair is unique, *i.e.* each peak in the first subset is paired with at most one peak from the second subset, and conversely. Iterations of this procedure are repeated on the remaining unpaired peaks, increasing the distance threshold until a maximum distance is reached. The steps above are repeated for each pair of symmetric scans in the dataset. This procedure allows fast identification of Friedel pairs in large datasets containing up to 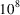 peaks, with relatively good completeness: the proportion of pairs matched typically reaches 70% to 90%, usually representing >80–90% of the total diffracting intensity.

### 2.3. Point-by-point phase mapping

#### 2.3.1. Mapping procedure

Once Friedel pairs have been identified and assigned to a pixel on a 2D grid, the dataset is processed pixel by pixel, in the same way as an electron backscatter diffraction map. The first step consists of creating a phase map, where each pixel is labeled with a phase. This step assumes that the space group and lattice parameters of all possible phases in the sample are known, allowing computation of theoretical diffraction angles 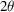 for each independent {*hkl*} family of crystal planes of each phase. In the following, 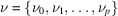 refers to the set of possible phase candidates, 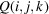 to an arbitrary pixel on the map, and 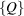 to the subset of peaks relocated within pixel *Q*.

Boolean selection masks are computed to assign diffraction peaks to each phase in ν, using a tolerance threshold around theoretical 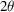 angles of individual {*hkl*} families. For a phase 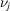, 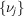 is the subset of peaks in this mask. A peak 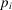 can belong to a multiple of these subsets, in the case of overlap between {*hkl*} rings from different phases. The sharp reduction of the 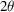 dispersion provided by the Friedel pair correction allows a reduction of the tolerance threshold, limiting the possibility of overlap between different {*hkl*} rings. In the easiest cases, where the sample contains only a few phases of high symmetry, overlaps are rare to non-existent. An unambiguous selection of peaks from the different crystallographic phases can then be achieved using these Boolean masks, from which a 2D phase label map is easily computed.

However, significant 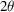 overlaps persist in more complex samples. In this case, treating overlapping conflicts pixel by pixel on the 2D grid can significantly improve the quality of the resulting phase map. Ambiguous situations where multiple phases overlap do not affect equally all regions of the sample. Overlaps are more prominent near grain boundaries than in grain centers and are also more common between phases sharing similar crystal structures than between phases of completely different symmetry and lattice parameters. Thus, confidence in phase assignment can vary greatly between different regions of the map. Pixel-by-pixel processing allows us to evaluate this confidence locally and to assign a label only to pixels for which a unique phase can be attributed with reasonable certainty. Phase labeling is done by summing the diffracting intensity of peaks in 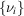 over pixel *Q* for each phase candidate in the set ν. The phase that collects the highest cumulated intensity over this pixel is retained, provided that the total peak count for this phase on pixel *Q* exceeds a minimal threshold 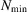, to avoid labeling pixels with only a few peaks. Hence, the best-matching phase 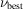 on pixel *Q* is the one maximizing the quantity
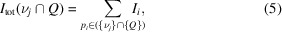
where 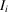 refers to the intensity of peak 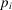. This process can be viewed as an approval voting procedure, where all peaks belonging to *Q* have to ‘approve’ or ‘disapprove’ each phase candidate 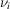, allowing selection of multiple candidates by each peak. This latter point is crucial, as it allows us to correctly take into account the preferences of each peak, accounting for existing ambiguities when two or more phases can match.

#### 2.3.2. Confidence assessment

Confidence in phase assignment may vary on each pixel of the map. To provide some quantification of how good is the match for a given phase on a given pixel, we introduce a confidence index, computed on each pixel. Considering a phase 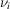 and a pixel *Q*, confidence in assigning 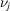 to *Q* is a combination of two different quantities: the completeness 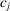 which quantifies the proportion of total diffracting intensity arising from *Q* that is collected by phase 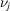, and the uniqueness of the selection 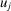 which quantifies the proportion of this diffracting intensity uniquely assigned to *Q*, *i.e.* not collected by any other phase. They are defined as follows: 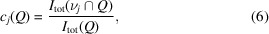
where 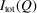 is the total diffracting intensity on pixel *Q*, and 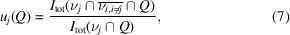
where the numerator corresponds to the integrated intensity of peaks in pixel *Q* that have been assigned to phase 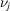, and only to 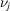. If the total peak count in 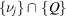 is below 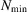, the pixel is kept unlabeled and both 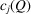 and 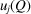 are set to zero. Assigning a phase with high confidence requires both high completeness and high uniqueness of the peak selection. Low completeness implies that the chosen phase matches poorly with the diffraction data. High completeness with low uniqueness implies that several phases possibly match, but cannot be easily discriminated.

However, the definition of completeness has one pitfall: assuming that the initial phase identification is complete, *i.e.* most of the peaks in 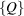 have been assigned to at least one subset 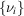, with *p* possible phase candidates, the lowest fraction of intensity collected by the best-matching phase 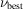 cannot be zero but will tend towards 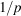 if the diffraction intensity arising from *Q* is uniformly distributed between the different phase candidates. Thus, the range of possible values for completeness depends on the number of phases being mapped and its lower bound tends to decrease with an increasing number of phase candidates. To avoid this effect, a normalization is introduced using the number of phases *p*: 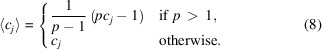
The confidence index 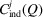 is then defined as the product of the two quantities 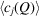 and 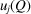: 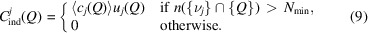
In the case 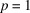, 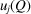 is trivial and takes only values 1 or 0 depending on whether the total number of peaks in 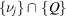 exceeds the threshold 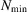. Thus, 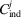 is merely a measure of completeness in this case.

The phase mapping process applied to test samples (see Section 3 below) produced maps with noticeable noise, emphasizing the necessity to implement a smoothing method to generate more realistic phase maps. This improvement can be realized by adopting a peak selection approach based on a moving kernel, rather than selecting peaks on individual pixels. The criteria for selecting the optimal phase and assessing confidence remain consistent with the definitions provided above, except that the selection domain *Q* encompasses an 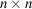 group of pixels centered on the pixel of interest, where *n* is an odd positive integer. This results in a better correlation between neighboring pixels, since the data used to determine the best-matching phase are partly shared between them.

### 2.4. Local indexing and grain mapping

Conventional processing of s3DXRD data involves indexing and grain mapping steps to produce a grain map. A grain is considered here as a continuous 3D domain consisting of a unique phase, over which lattice orientation is constant or varies smoothly within a limited range. It is characterized by a unit-cell matrix, formed by the real-space lattice vectors in sample coordinates. This matrix is usually denoted as a product 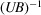, where *U* is the crystal rotation matrix giving the grain orientation in the sample frame and *B* the matrix formed by the reciprocal unit-cell vectors (Busing & Levy, 1967 ▸). The rows of the matrix are the real-space lattice vectors.

Indexing aims to identify grains in the dataset by finding clusters of diffraction vectors in reciprocal space (**g** vectors) compatible with a unique orientation *U*, assuming *B* constant and equal to a known reference cell. Each unit-cell matrix found corresponds to a grain candidate of orientation *U*. Once this step has been done, a refinement procedure is run to exclude peak outliers, remove spurious grains, refine *B* and fit the grain center of mass. The grain shapes are then reconstructed using the filtered back-projection of the sinogram of peaks assigned to each grain. This procedure usually works well for samples that have experienced small deformations. With increasing strain, the assumption that lattice orientation is close to constant over a grain becomes less and less valid, as large internal misorientation can occur. Moreover, this procedure is done simultaneously over an entire 2D slice of the sample, with potentially hundreds to thousands of grains to match at a time, increasing both computation time and the probability of orientation overlaps between different grains. Thus, indexing becomes increasingly challenging with increasing strain and sample size.

Starting from the labeled pixel map with diffraction peaks assigned to each pixel, the problem can be seen from a different perspective: each pixel is characterized by a unique matrix of real-space unit-cell vectors, which is fitted using the set of diffraction peaks assigned to it. The fitting process is done separately for each phase using indexing functions from *ImageD11*. If no compatible unit-cell vector matrix is found, or if too few peaks match with the best matrix, the pixel remains unindexed.

Similarly to phase mapping, the local indexing procedure tends to generate noisy and incomplete maps when applied pixel by pixel. Again, this issue is solved by introducing a kernel selection approach of peaks used to fit the local unit-cell vector matrix. However, this comes at the expense of a rapid increase in computation time with increasing kernel size, because the number of **g** vectors to fit for each pixel scales with the square of the kernel size *n*.

The output of the local indexing procedure is a map where orientation is defined on a per-pixel basis, but no grains have been defined yet at this stage. A grain map can be obtained *a posteriori* by grouping pixels together. Various methods can be used. The most straightforward is to cluster pixels by orientation. First, each local orientation is mapped to the symmetry-reduced fundamental zone of orientation space corresponding to the symmetry of the phase being mapped. Then, a clustering algorithm is run to find groups of pixels with similar orientation (Johnstone *et al.*, 2020 ▸). Once pixels have been clustered, grain masks are defined, and mean grain lattice vectors are fitted using the full set of peaks collected over the whole grain masks. This approach works as long as orientation clusters corresponding to different grains are well separated, but it becomes ineffective for highly strained, highly textured samples containing many grains with overlapping orientation distribution functions. More advanced grain reconstruction algorithms may be used in this case, such as the fast multi-scale clustering algorithm implemented in *MTex* (McMahon *et al.*, 2013 ▸).

## 3. Applications

### 3.1. Sample description

**Figure 4:**
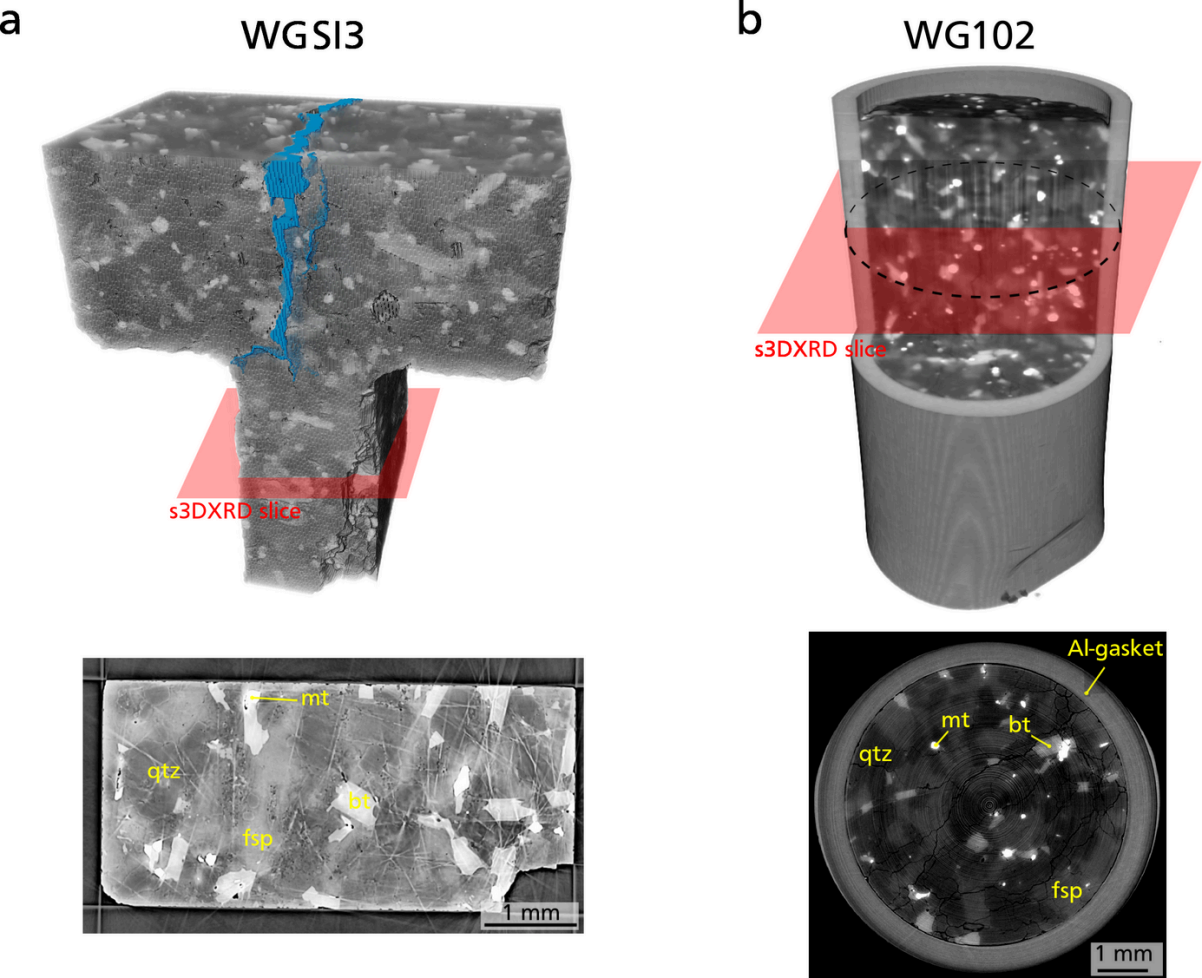
Microcomputed X-ray tomography (µCT) reconstructions of Westerly granite samples WGSI3 (*a*) and WG102 (*b*). The red surface shows the approximate position of s3DXRD scans in each sample. The blue domain in sample WGSI3 corresponds to open fractures with an aperture larger than 12.4 µm. The lower panels show the corresponding µCT slice, with different minerals labeled. bt: biotite; fsp: feldspar (orthoclase or oligoclase); mt: magnetite; qtz: quartz.

The workflow described above has been applied to two samples of Westerly granite (WG102 and WGSI3). This rock is a fine-grained equigranular granite quarried in the Westerly area (Rhode Island, US) and has been used for a long time as a reference material for rock physics studies (Brace & Byerlee, 1966 ▸; Lockner, 1998 ▸). Westerly granite is actually a quartz monzonite to granodiorite, mainly composed of quartz, potassic feldspar (microcline) and plagioclase (oligoclase) in similar proportions, in addition to subordinate amounts of biotite, white mica, and magnetite and hematite iron oxides (Fairbairn, 1951 ▸). The two samples WGSI3 and WG102 were recovered from dynamic loading ‘shock’ experiments performed using split-Hopkinson pressure bars, such that they have been damaged dynamically (Doan & Gary, 2009 ▸). Therefore, they feature high internal brittle damage that consists of multiple tensile cracks, mainly opened along a direction orthogonal to the propagation of the compression wave. These two samples were imaged after the shock experiment using X-ray microtomography on beamline ID19 (ESRF), with a voxel size of 6.2 µm. Sample WG102 [Fig. 4 ▸(*b*)] is a 5 mm-diameter by 10 mm-height core, which was placed in a 0.5 mm-wide gasket made of commercial aluminium alloy to avoid complete pulverization during the shock. WGSI3 [Fig. 4 ▸(*a*)] is *ca* 5 mm wide. It has a more complex T-shape than WG102 and was not placed in a metal gasket, but nevertheless survived the shock experiment without being pulverized so that the entire damaged sample could be recovered. These two samples represent the archetypal materials that geologists need to investigate. They feature almost all the different types of complexities prevalent in geological samples, including large sample sizes, the presence of multiple low-symmetry phases and substantial internal damage. However, these samples lack significant ductile deformation. The aluminium gasket surrounding sample WG102 provides in addition a relevant example of a ductile material that features large plastic strain.

Diffraction data were acquired at beamline ID11 of ESRF, using a pencil beam scanning 3DXRD procedure. One slice of sample WGSI3 was scanned at a low resolution (50 µm beam size) on the 3DXRD experimental station of ID11, equipped with a FReLoN 2K detector (47 µm pixel size). One slice of sample WG102 was scanned at a higher resolution (10 µm beam size) on the nanofocus station, equipped with an Eiger2 X CdTe 4M detector (75 µm pixel size). Details about X-ray beam characteristics and scanning procedure are summarized for each sample in Table 1 ▸. Examples of raw diffraction frames for these two samples are provided in the supporting information. Calibration and pre-processing of diffraction frames were performed using *ImageD11* (Wright, 2024 ▸). Calibration of detector geometry (distance, center, wedge and tilt) was performed before each scan using a NIST reference CeO_2_ powder and a piece of a pristine, hydrothermal quartz monocrystal sampled in the massif of Belledonne (western Alps).

### 3.2. Matching Friedel pairs

**Figure 5:**
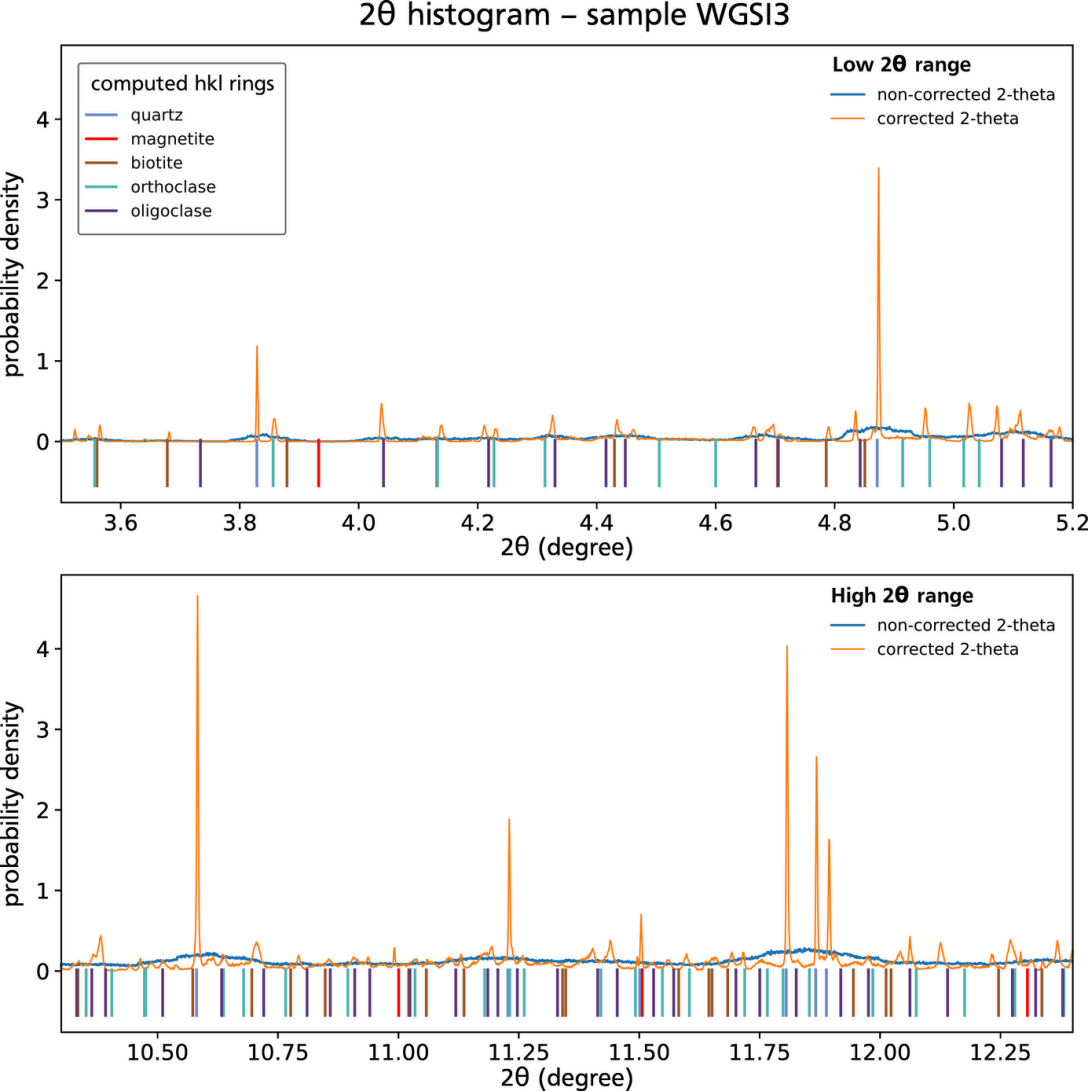
Probability density of 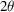 data for raw (blue) versus corrected (orange) diffraction peaks for sample WGSI3, in the low 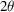 (top) and the high 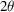 (bottom) range. The Friedel pair geometric correction results in a sharp decrease of 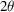 broadening, revealing Bragg peaks corresponding to the different crystal phases in the sample, which were completely blurred in the non-corrected dataset. Vertical ticks on the bottom of each plot show the position of computed Bragg peaks for the different phases.

Friedel pairs were matched for each dataset, using the algorithm introduced in Section 2. The proportion of matched pairs is fairly reasonable, around 71% for WG102 and 76% for WGSI3. The geometric correction using Friedel pairs was applied to recover the offset-corrected 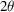 angles and relocate the diffraction source in the sample. The effect of this correction on 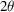 angles appears clearly when plotting the histogram of 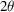 for corrected and uncorrected peaks (Fig. 5 ▸). The spread of 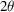 angles is greatly reduced and these angles match with pre-computed 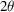 positions of theoretical {*hkl*} rings, meaning that accurate measurements of the real 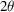 angle are obtained for each crystal plane.

**Figure 6:**
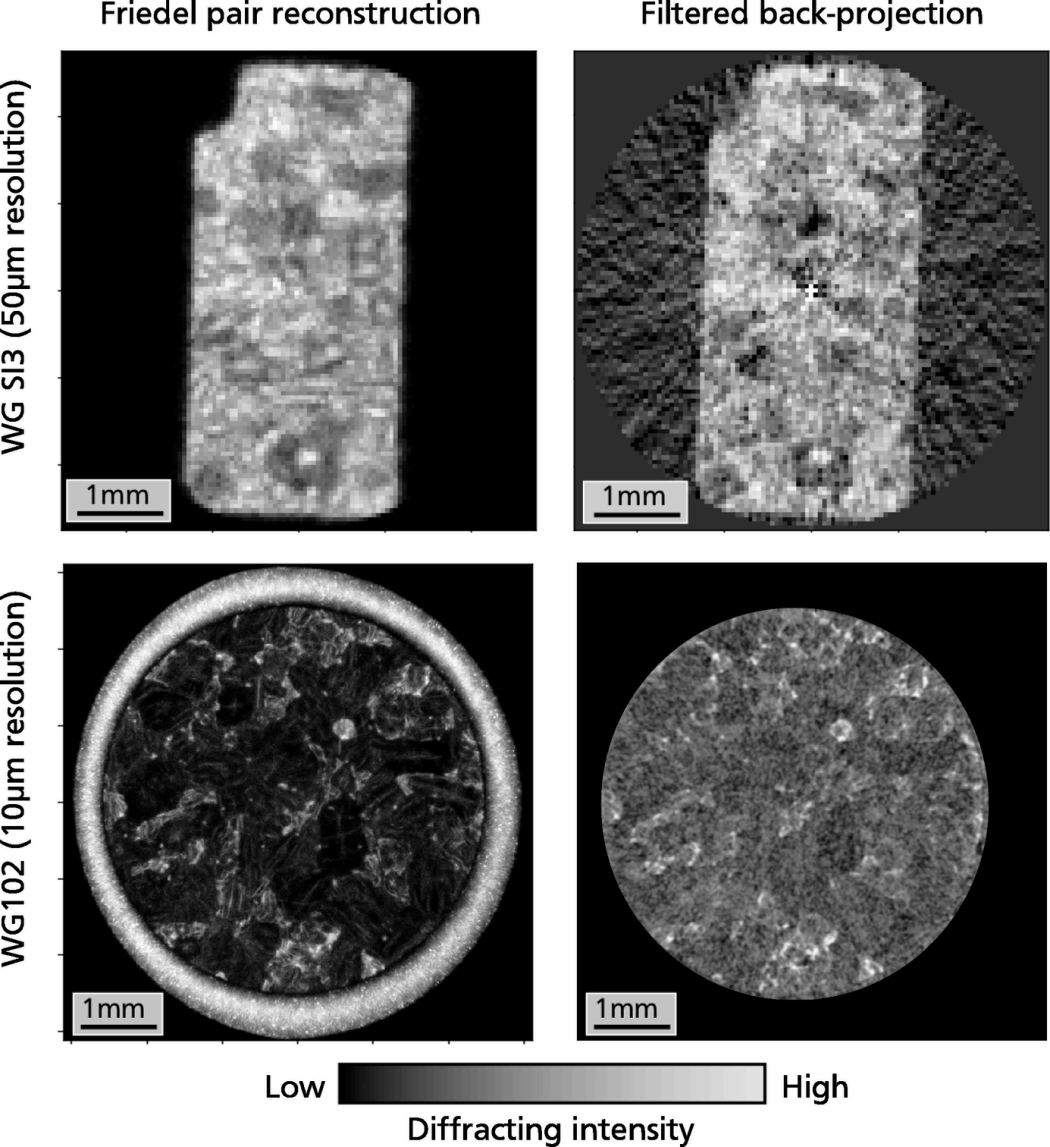
Comparison of 2D slice reconstruction using the Friedel pair method and the filtered back-projection method, for the two samples WGSI3 (low scanning resolution) and WG102 (high scanning resolution). The gray color scale is indicative of the total diffraction intensity arising from each pixel (brighter color = higher intensity). The filtered back-projection algorithm performed very poorly with the aluminium jacket surrounding sample WG102, so the filtered back-projection reconstruction (bottom right) has been obtained after filtering out the diffraction peaks of this metal.

Diffraction image reconstructions of the scanned sample slices are obtained from the relocated peak sources by plotting a 2D histogram of cumulated diffraction intensity on each pixel [Fig. 6 ▸(left)]. The reconstructions obtained through this method exhibit a good correlation with tomographic reconstructions achieved using the filtered back-projection algorithm on the sinogram of total diffracted intensity [Fig. 6 ▸(right)]. On these specific samples, the Friedel pair reconstruction appears to outperform the filtered back-projection method, generating fewer artifacts. Specifically, the presence of the Al gasket surrounding granite in sample WG102 resulted in pronounced artifacts in the reconstruction of the granite core. This was likely caused by the much higher diffracting intensity of the metal gasket compared with the rock sample. To produce a filtered back-projection reconstruction wherein different grains can be distinguished, the diffraction peaks of aluminium had to be cropped out, while the Friedel pair method easily reconstructed the entire sample slice, including the aluminium gasket.

### 3.3. Phase mapping

Phase maps were obtained considering a simplified mineral assemblage for granite in both samples, consisting of quartz, oligoclase, orthoclase, biotite and magnetite. This mineralogical composition excludes any accessory or secondary alteration phases that may be present in minor proportions in the rock, which are likely to be insignificant in the total diffracting intensity. Oligoclase and orthoclase represent idealized compositions of the two types of feldspar, respectively, plagioclase and potassic feldspar, occurring in the granite, which are here considered as phases of fixed composition rather than solid solutions. In addition, face-cubic-centered aluminium was included for the gasket in sample WG102.

**Figure 7:**
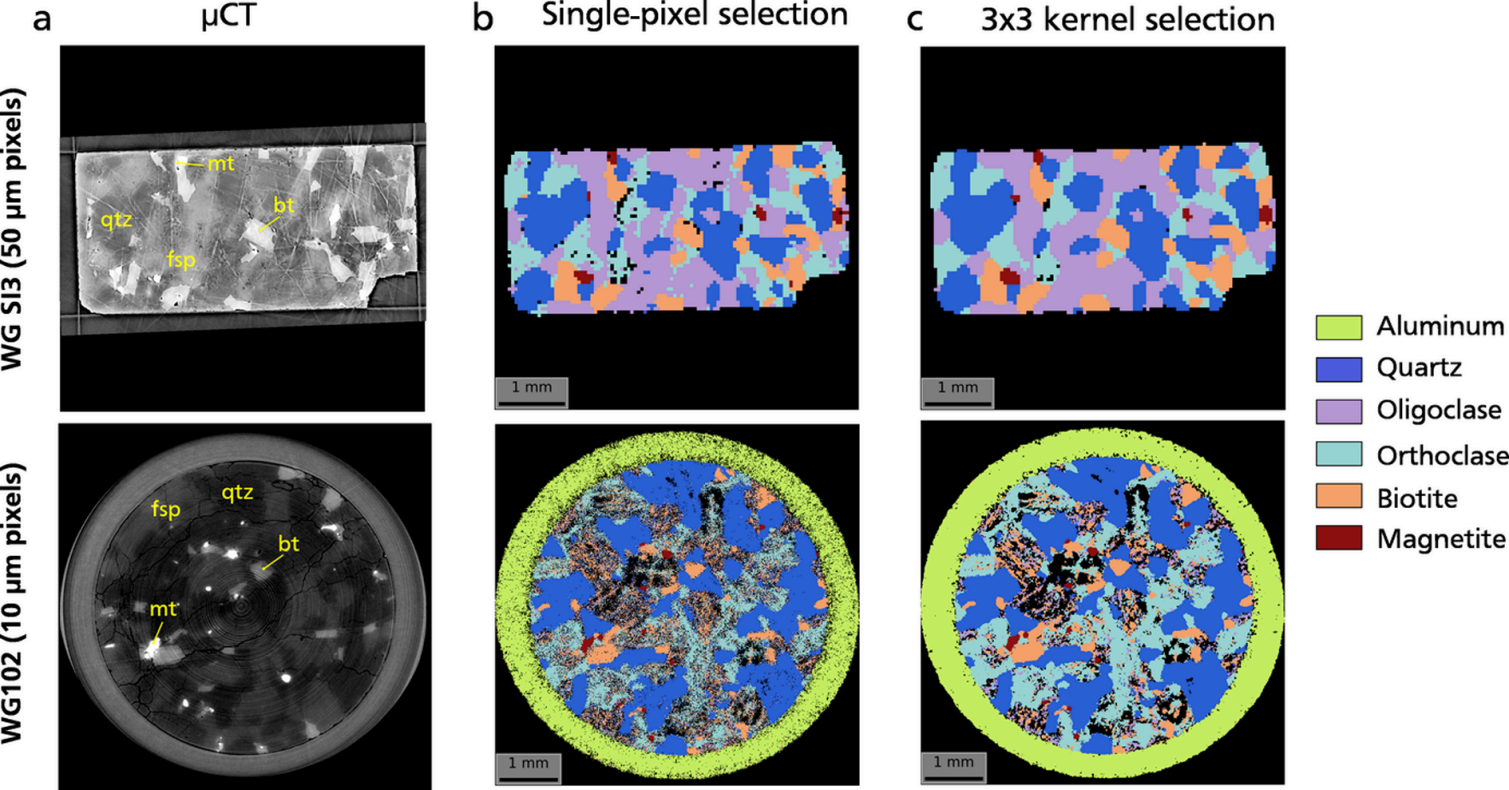
Phase maps for samples WGSI3 and WG102, placed next to µCT images (*a*) of the same slice for comparison. The different minerals considered for phase mapping are listed on the right. Corresponding space groups and lattice parameters are summarized in Table 2 ▸. Two mapping options are shown, using either single-pixel selection (*b*) or kernel selection (*c*). With single-pixel selection, the best-matching phase on a pixel is assessed considering only diffraction peaks assigned to this pixel. With kernel selection, the best-matching phase on a pixel is assessed considering all peaks belonging to a 3 × 3 square of pixels around the central pixel, resulting in smoother and more complete phase maps.

The resulting phase maps [Figs. 7 ▸(*b*), 7 ▸(*c*); Table 2 ▸] are plotted next to X-ray microtomography (µCT) reconstructions of the same 2D slice [Fig. 7 ▸(*a*)] for both samples. Gray tones on the µCT images represent different minerals and exhibit a strong correlation with the phase maps. Specifically, Fe-bearing phases such as biotite and magnetite, which appear the brightest and are easily identifiable in µCT, demonstrate a notable overlap with the s3DXRD phase maps. However, achieving perfect registration between the µCT and 3DXRD images proved challenging. This is because the µCT scanning was conducted at a higher resolution (6.2 µm voxel size) than the s3DXRD, resulting in imperfect matching of the sampled volumes for a given slice. Additionally, the s3DXRD and µCT data were acquired on different instruments, with sample mounting leading to slightly different rotation axis directions in the sample, further complicating image registration.

Although the phase maps in Fig. 7 ▸(*b*) appear quite satisfactory to first order, they still exhibit noticeable noise and incompleteness, with many pixels remaining unlabeled. This is particularly evident for phases with low symmetry, such as oligoclase and orthoclase, and at high spatial resolutions (sample WG102). To improve phase maps, we used the kernel mapping procedure, using a 3 × 3 kernel. This step led to significant improvement in terms of both noise reduction and completeness of the phase maps [Fig. 7 ▸(*c*)], although the high-resolution map (WG102) is still not completely satisfactory. However, mapping artifacts are primarily observed in low-symmetry phases, whereas the maps of higher-symmetry phases (quartz, magnetite, aluminium) are of much better quality.

**Figure 8:**
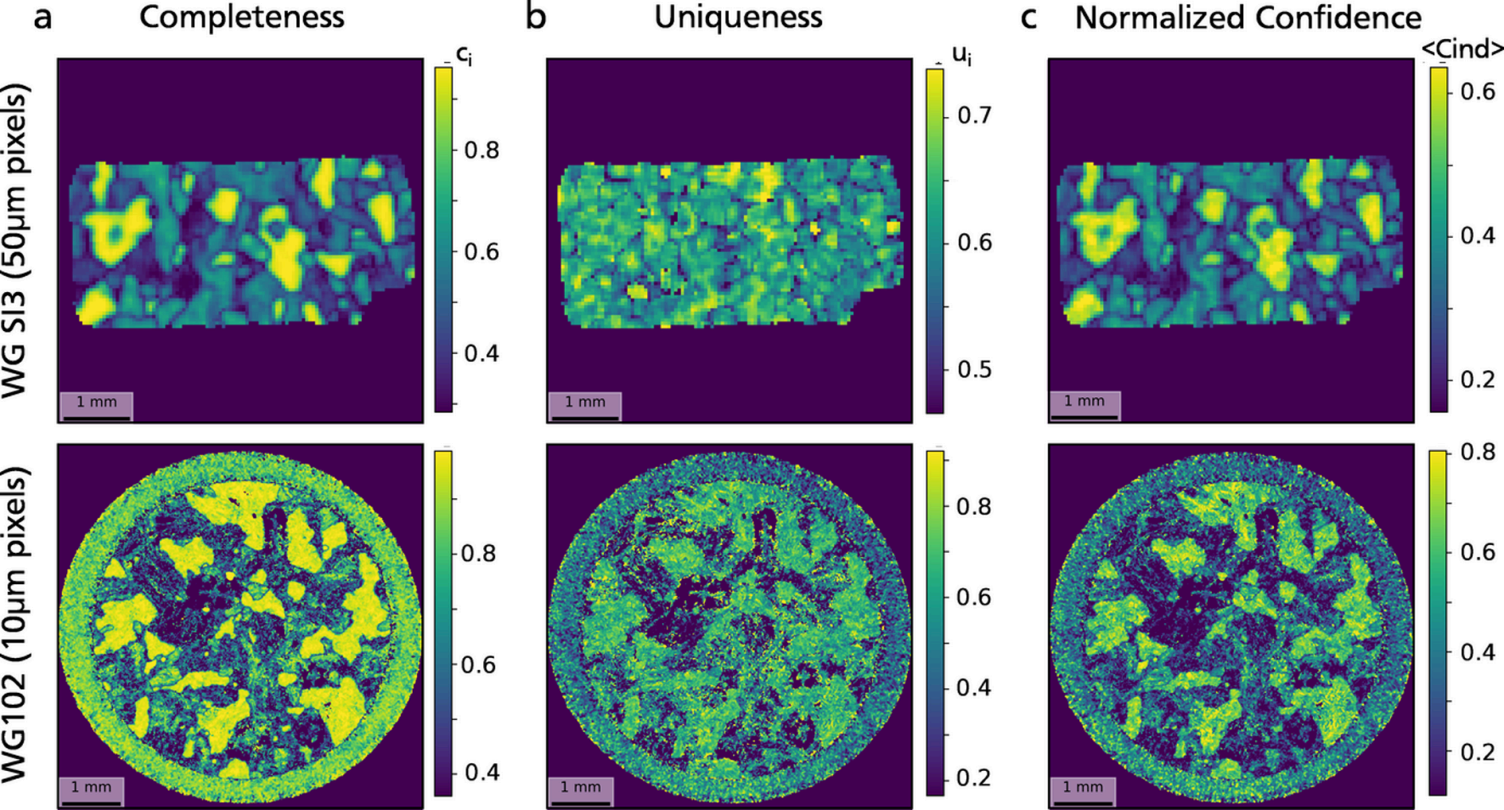
Completeness (*a*), uniqueness (*b*) and normalized confidence index (*c*) maps for samples WGSI3 and WG102, computed using a 3 × 3 kernel peak selection. These indexes are used to evaluate confidence in phase assignment over each pixel. See Section 2 for more details.

As depicted in Fig. 7 ▸, the quality of phase maps varies significantly from phase to phase, underscoring the disparity in confidence levels regarding phase assignment. This confidence can be quantified using the uniqueness, completeness and normalized confidence indexes defined in Section 2. These three criteria are plotted in Fig. 8 ▸ for the phase maps obtained using the 3 × 3 kernel method [Fig. 7 ▸(*c*)].

Completeness exhibits substantial variation across different phases, with values close to 1 for high-symmetry phases like quartz and magnetite, and declining significantly for phases with lower symmetry, sometimes dropping below 0.3. Grain boundaries are notably discernible on the maps [Fig. 8 ▸(*a*)] as regions with lower completeness. Uniqueness, on the other hand, shows a more uniform distribution and is less phase dependent, albeit still influenced by grain boundaries [Fig. 8 ▸(*b*)]. However, it rarely exceeds values above 0.7, indicating significant overlapping in scattering angles between different phases. The normalized confidence index, computed as a combination of these two values [Fig. 8 ▸(*c*)], consequently ranks highest for quartz and magnetite, is lower for Al and biotite, and is notably poor for the two feldspars (oligoclase and orthoclase). This disparity explains the high levels of noise and incompleteness exhibited by these two phases.

### 3.4. Lattice orientation fitting and grain mapping

**Figure 9:**
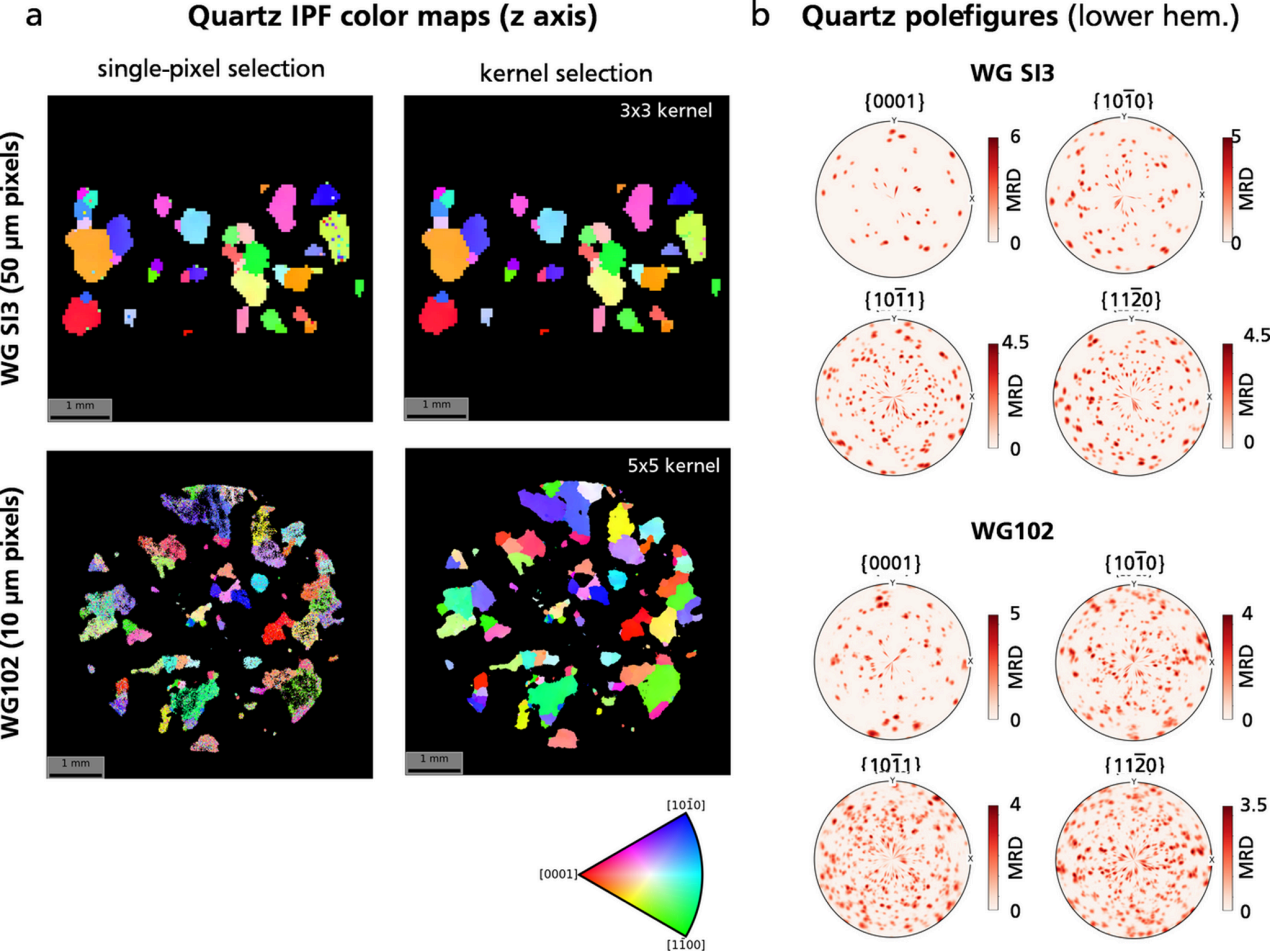
Orientation of quartz pixels for samples WGSI3 and WG102, shown as inverse pole figure (IPF) color maps relative to the vertical *z* axis in the sample reference frame (*a*) and as lower-hemisphere pole figures for a selection of Miller indices (*b*). Orientation obtained using peak selection over single pixels generates noisy maps. Peak selection using an 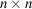 kernel significantly improves the quality of orientation maps, especially for sample WG102. Computation with kernel selection took *ca* 2 h for sample WG102 and *ca* 5 min for sample WGSI3, using a full node (40 cores) of the Nice cluster at ESRF. Orientation-to-color mapping and pole figure plots were obtained using the *orix* library (Ånes *et al.*, 2024 ▸).

Point-by-point fitting of the unit-cell vector matrix is illustrated for quartz, which produced the highest confidence score during phase mapping and is present in significant proportions in the granite. Quartz unit-cell matrices were computed for samples WGSI3 and WG102, utilizing either the single-pixel selection method or a kernel selection. For sample WG102, a larger 5 × 5 kernel was necessary to achieve a satisfactory level of smoothing and ensure complete mapping of quartz orientation. The resulting pixel orientation maps are shown in Fig. 9 ▸(*a*) for both samples, illustrating the results of the single-pixel method (left panels) and the kernel method (right panels).

**Figure 10:**
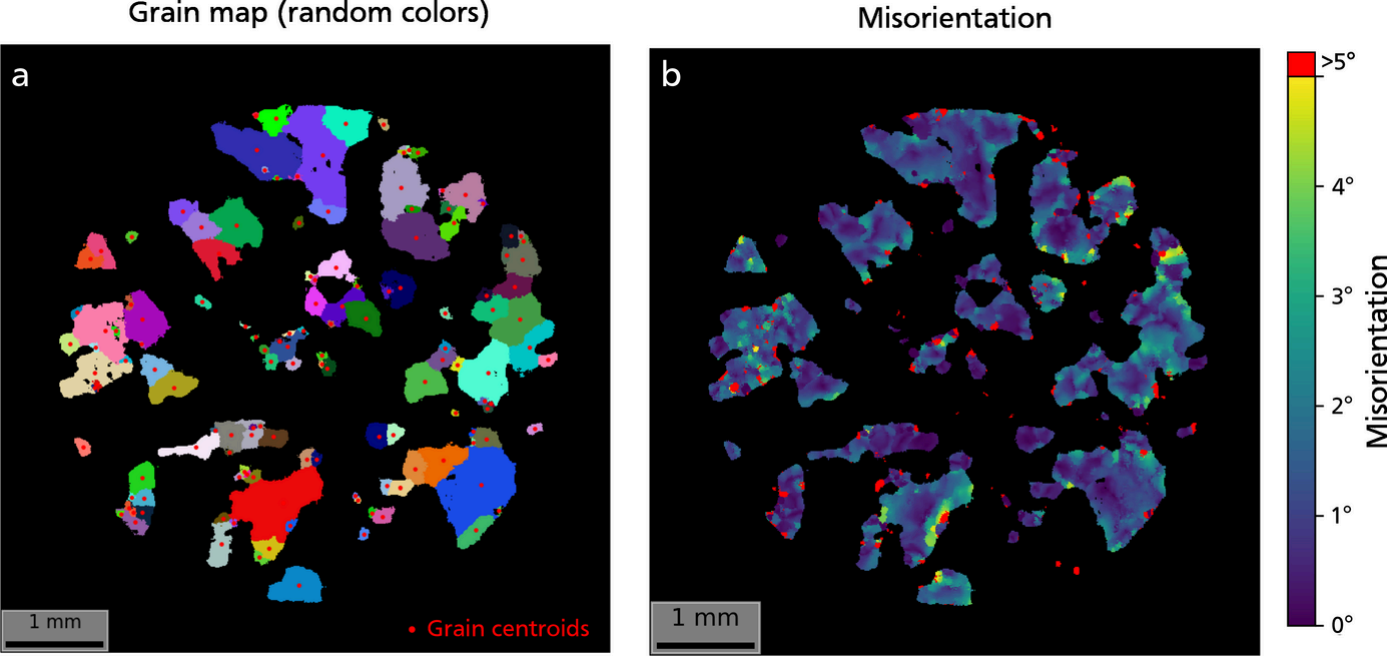
Grain map of sample WG102, obtained by clustering pixel orientation in the symmetry-reduced fundamental zone (Johnstone *et al.*, 2020 ▸). (*a*) Grains are colored by random colors. Red dots correspond to the grain centroids. (*b*) Grain internal misorientation in degrees. Red areas correspond to high misorientation (≥5°) and highlight unreliable areas that were not correctly identified as separate grains.

The quartz pixel orientation distribution function plotted for a selected set of 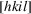 Miller indices [lower-hemisphere pole figure, Fig. 9 ▸(*b*)] reveals distinct clusters, corresponding to different grains within the scanned volume. However, orientations have not been defined yet at the grain level, which requires grouping of contiguous pixels sharing similar orientations. Considering the nearly discrete nature of the quartz orientation distribution function, a basic clustering approach in orientation space is sufficient to yield a satisfactory grain map. The grain mapping process is shown for sample WG102. The density-based spatial clustering of applications with noise (DBSCAN) algorithm from *scikit-learn* (Pedregosa *et al.*, 2011 ▸) was employed to cluster quartz pixels by orientation in the symmetry-reduced fundamental zone (point group 3*m*). A total of 136 grains were identified [Fig. 10 ▸(*a*)]. The mean grain orientation and lattice parameters were then refined using the full set of **g** vectors assigned to each grain. The misorientation, as computed in Fig. 10 ▸(*b*), represents the deviation in degrees between the pixel orientation and the mean grain orientation. This map exposes significant internal orientation gradients and sub-grains with misorientations of up to 5°, which were challenging to discern on the orientation color map (Fig. 7 ▸). Areas highlighted in red denote >5° misorientation, pinpointing a few problematic sub-grains that were not accurately identified as distinct grains.

**Figure 11:**
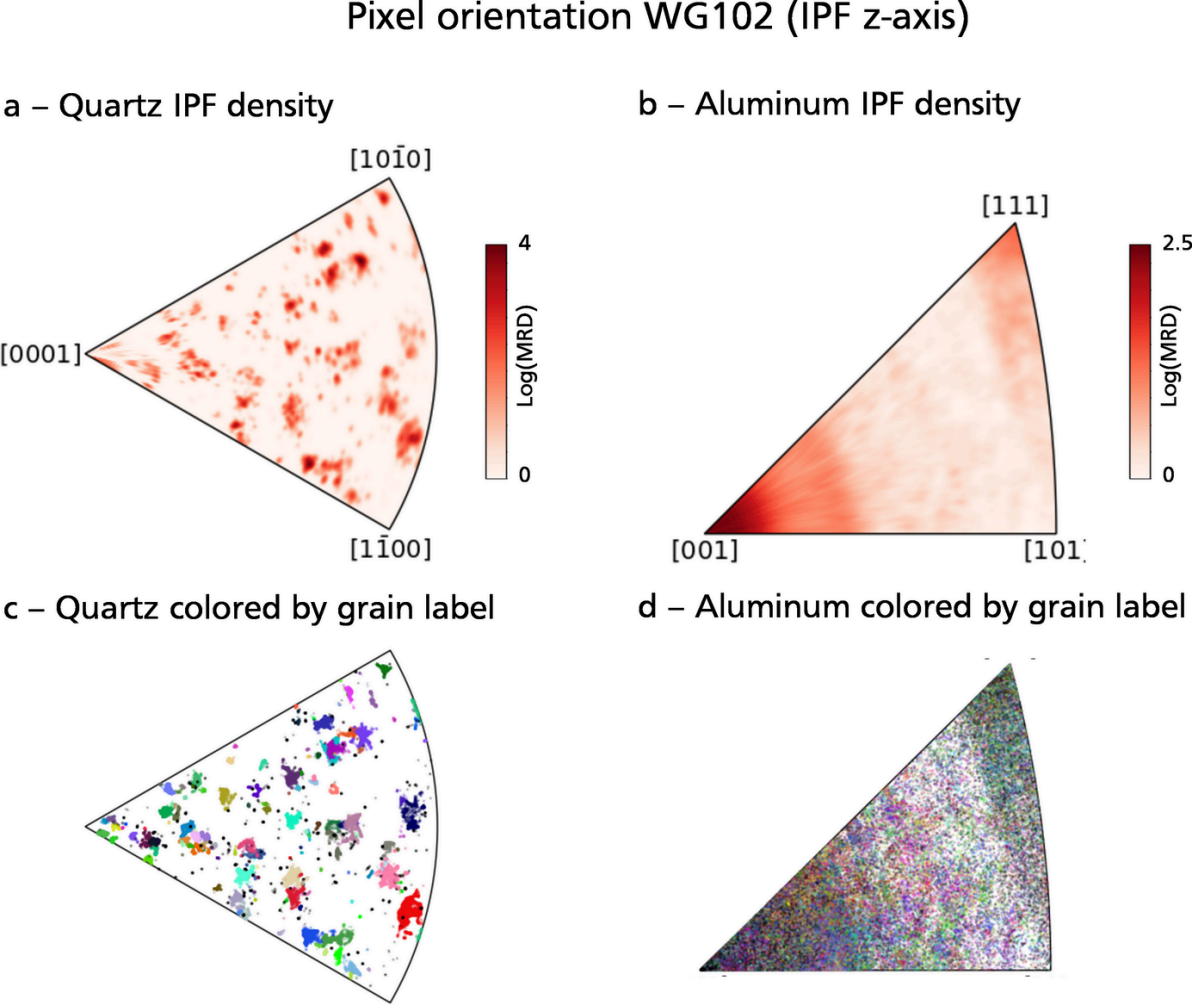
Pixel orientations and results of DBSCAN clustering for sample WG102, plotted in inverse pole figure space relative to the *z* axis. Inverse pole density for quartz (*a*) and aluminium (*b*) pixels. MRD: multiple of a random (uniform) distribution. Scatter plot of pixel orientations colored by grain label, for quartz (*c*) and aluminium (*d*). Each color corresponds to a cluster identified by the DBSCAN algorithm and matches with the grain colors in Fig. 10 ▸. For quartz, most of the orientation clusters visible in the inverse pole figure were identified correctly. For aluminium, the lack of well defined orientation clusters resulted in poor outcomes for grain mapping.

**Figure 12:**
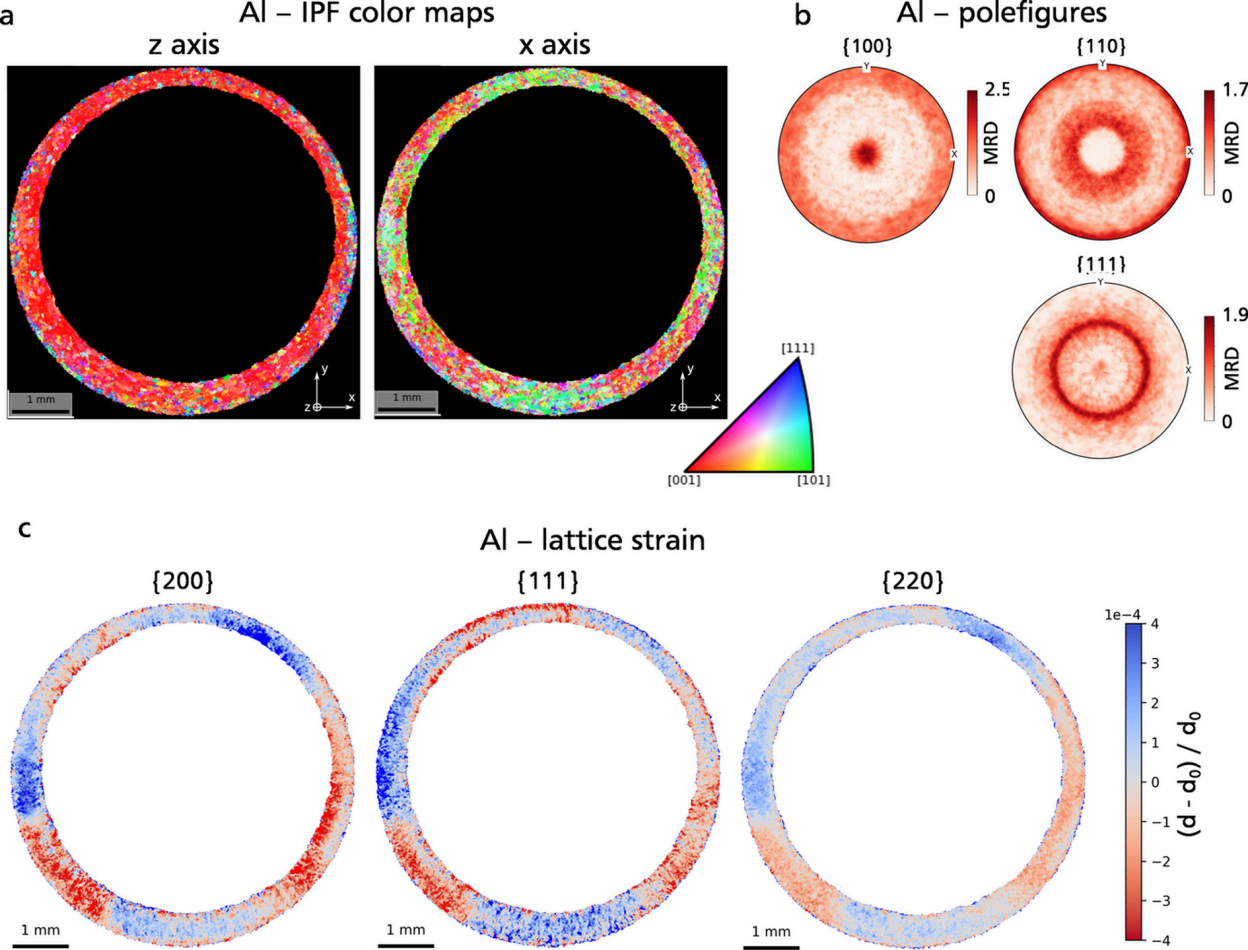
Orientation and strain maps for the Al gasket surrounding a sample similar to WG102. (*a*) IPF color maps of pixel orientation relative to the *z* and *x* axes in the sample reference frame. (*b*) Pixel orientation (lower hemisphere) for a selection of Miller indices. (*c*) Strain maps computed for several {*hkl*} diffraction rings. *d* is the *d* spacing for crystal planes, and 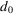 corresponds to the median *d* spacing for all diffraction peaks belonging to the selected {*hkl*} family over the whole sample. Strain is given in engineering convention (negative strain for shortening) and is expressed in a direction orthogonal to the orientation of the selected {*hkl*} crystal planes.

The orientation clustering approach proved effective for quartz because there are only a limited number of grains forming well separated clusters in Euler angle space [Fig. 11 ▸(*a*)]. However, grain mapping is expected to become increasingly difficult with increasing plastic strain and decreasing grain size. A particularly challenging case is provided by the Al gasket surrounding the granite sample. This gasket was made by drilling and cutting a bar of commercial aluminium alloy. Hence, it features a fine-grained (grain size < 50 µm) and strongly anisotropic texture, with a strong preferred orientation of {100} faces orthogonal to the gasket. This texture is shown in Figs. 12 ▸(*a*) and 12 ▸(*b*) for a sample similar to WG102, in which the Al gasket features radial variations – lacking in WG102 – of crystal orientation around the gasket. As visible on the inverse pole density map [Fig. 11 ▸(*b*)], the aluminium orientation distribution function shows a much broader range of orientation than the quartz orientation distribution function, leading to highly unsatisfactory results of DBSCAN clustering [Fig. 11 ▸(*d*)].

## 4. Discussion

### 4.1. Advantages of Friedel pairs

Relocation of diffraction peaks within the sample using Friedel pairs allows the processing of s3DXRD data on a per-pixel basis, which has several advantages over classical indexing procedures.

First, there is no need to process the entire map. Once diffraction peaks have been relocated in the sample, it becomes possible to isolate peaks from a smaller sub-domain for subsequent processing. This approach proves particularly beneficial for fine-tuning tolerance parameters utilized in phase mapping and local indexing before embarking on processing the entire map. Additionally, when the region of interest (ROI) constitutes merely a fraction of a larger sample, acquiring data across its entire width is unnecessary. Instead, the sample can be precisely positioned to align the ROI with the rotation center of the sample stage. Then, the point-focused beam scanning procedure is conducted over a limited range of *y* positions, encompassing the width of the ROI. Friedel pairs relocated beyond the boundaries of the ROI are subsequently filtered out, leaving only the data pertinent to the ROI. This method improves the flexibility of the s3DXRD technique, facilitating the straightforward selection of sub-volumes without using slits to physically filter the range of scattering angles reaching the detector.

Another advantage lies in the ability to derive local information from diffraction data without requiring indexing and grain mapping steps. Each diffraction peak is directly relocated within the sample before indexing, enabling certain properties to be mapped on a per-pixel basis, even in cases where local indexing fails. This point is illustrated using the Al gasket described in Fig. 12 ▸, which displays a strong preferred orientation of aluminium grains, presumably resulting from the large strain caused by extrusion during manufacturing. It may be interesting to quantify residual strain and stress and map their distribution within the gasket. However, the metal constituting the gasket is too deformed to consistently identify grains from the pixel map [Fig. 11 ▸(*b*)]. Nevertheless, orientations retrieved on a per-pixel basis appear to be consistent, although resulting in a visually noisy map [Fig. 12 ▸(*a*)]. Furthermore, the unit-cell volume computed using the lattice vectors fitted on each pixel exhibits variations of over 15% across the sample, corresponding to unrealistic levels of elastic strain. This discrepancy arises from the very elongated shape of Al peaks on the detector and indicates that the unit-cell matrix fitted on each pixel is unreliable and unsuitable for accurate strain calculations.

While this approach fails, an alternative method involves selecting peaks from a single {*hkl*} family and calculating the {*hkl*} lattice strain 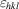 as follows: 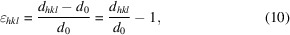
where 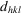 is the lattice spacing for the selected peaks and 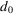 is the lattice spacing of an unstrained reference. It is computed for each peak belonging to the selected {*hkl*} group. Then, the median strain is determined for each pixel, generating a map of strain variations across the sample. This process only requires knowledge of which diffraction peaks are assigned to a specific pixel, obviating the need for fitting a lattice vector matrix. The resulting strain maps, depicted in Fig. 12 ▸(*c*) for the {200}, {111} and {220} families of crystal planes, take the median {*hkl*} *d* spacing over the entire sample as the 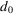 reference. The strain fluctuates from *ca*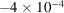 to 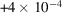 and exhibits a similar spatial distribution across different {*hkl*} families [Fig. 12 ▸(*c*)], with certain regions of the gasket experiencing compression (red) and others undergoing tension (blue). Interestingly, this spatial distribution correlates with the lattice orientation *U* fitted for each pixel. This result might be due to the elastic anisotropy of Al crystals (Kube, 2016 ▸), which hence respond differently to stress depending on orientation.

However, the interpretation of these maps in terms of strain field in the sample reference frame is not completely straightforward: 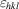 corresponds to strain orthogonal to a given set of lattice planes in the crystal. Therefore, some constraints on the crystal orientation *U* are needed to relate these values to the strain in the sample reference frame. Moreover, the set of reflections selected to compute the median 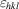 over each pixel corresponds to the whole {*hkl*} family, including all possible (*hkl*) planes equivalent by symmetry. If the multiplicity is greater than two, *i.e.* the {*hkl*} family contains more than just the (*hkl*) and (*h**k**l*) planes, 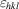 will be the median of strain along different directions of the crystal equivalent by symmetry. This can be difficult to interpret intuitively, except in some specific cases. For example, for a cubic system, 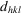 is directly related to the unit-cell parameter *a*: 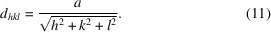
Therefore, 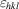 can be related to volumetric strain 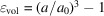: 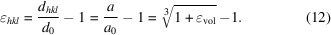
This analysis assumes however that strain is small and the unit cell remains approximately cubic. For crystals of lower symmetry, the relation with intuitive strain components may not be as straightforward.

### 4.2. Current limitations and pitfalls

#### 4.2.1. Completeness and robustness of Friedel pair matches

The Friedel pair matching algorithm has been optimized to reduce computing time so that very large datasets containing several tens to hundreds of millions of peaks – typical figures for s3DXRD – can be processed. However, this to some extent comes at the expense of completeness and robustness of pair identification. The proportion of Friedel pairs matched usually ranges between 70% and 90% depending on the sample. Above a certain point, increasing the ‘max distance’ parameter, which controls the maximum distance between two peaks forming a pair in the 4D search space, does not improve this value any further, and mostly results in the random pairing of residual peaks. The presence of residual single peaks probably results from the fact that possible pairs are searched only in symmetrical scans acquired at *y* translations 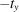 and 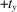 of the sample relative to the central position [Fig. 3 ▸(*a*)]. Thus, if the beam is not perfectly aligned with the rotation center at the central position, some pairs will be missed because sample illuminations at 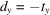 and 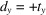 will not be perfectly identical. This issue is likely to be increasingly problematic with increasing scanning resolutions, especially below sub-µm beam sizes. In such cases, very precise positioning of the sample is required to ensure the symmetry of the 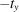 and 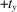 scan pairs. Slight inaccuracies in motor positioning or small displacement of the sample during scanning (if it is not correctly fixed on its holder) will result in significant decorrelation of scans, hindering the correct identification of pairs.

More pairs could probably be recovered by extending the search to neighboring scans, *i.e.* looking at peaks in scans 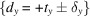 to match with the peaks in 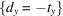. However, the proportion of pairs recovered is usually high enough to allow robust mapping of phase and orientation over each pixel. Moreover, the Friedel pair match is usually better for the strongest peaks, *i.e.* unpaired peaks are more frequently weak-intensity peaks. Hence, the fraction of total intensity matched during the Friedel pair search is usually high, typically above 80% to 90%. Therefore, further improving the completeness of Friedel pair matching does not appear to be very critical.

Robustness can also be an issue. The nearest-neighbor search can yield ambiguous results if peaks arising from different grains are very close to each other on the detector, making the presence of false-positive pairs unavoidable. A few can be spotted in Fig. 3 ▸(*b*), but they are rare, representing only a minor proportion of peaks in the plot. A possible improvement could be the implementation of a robustness criterion, which would quantify the probability of a given pair being a false positive, and could be used to filter out spurious Friedel pairs. This criterion should depend on the distance between paired peaks (the lower, the better), as well as the number of other peaks in the close neighborhood (the lower, the better) in the 4D search space.

Nevertheless, the false-positive pairs are already removed to some extent during subsequent processing steps. Indeed, 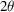 angle correction and peak relocation in the sample are likely to be unreliable for such pairs. Some of them are relocated outside of the sample and are readily removed. Some others are also probably filtered out during the phase labeling step because they have an incorrect 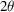 angle, which does not match with any possible phase candidate. Finally, some unreliable peaks are also removed during local indexing, which involves a refinement stage where peak outliers are removed. Therefore, the presence of a small proportion of false-positive Friedel pairs in the dataset does not seem to be a critical issue for further point-by-point mapping of phase and orientation.

#### 4.2.2. Inaccuracy of the point-by-point fit method

The pixel-fit method introduced in the present study is analogous to the point-fitting method proposed by Hayashi *et al.* (2017 ▸). In the latter, the crystal structure (unit-cell vector matrix) undergoes refinement on a point-by-point basis, using the subset of reflections for which the designated point falls within the path of the incident beam. Our method operates on a similar principle, but the selection of the subset of reflections used to fit the lattice state at a specific point is based on the back-projection of diffraction peaks using Friedel pair properties. This step represents an improvement since it enables the direct selection of reflections originating from a single grain in the sample. In contrast, the method proposed by Hayashi *et al.* (2017 ▸) selects all reflections arising from rays intersecting the point of interest at a particular 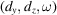 scanning position, necessitating subsequent filtering to retain only the relevant diffraction peaks.

However, a major drawback of this approach stems from the erroneous assumption that diffraction events originate from individual points within the sample. As highlighted by Henningsson *et al.* (2020 ▸) and Henningsson & Hendriks (2021 ▸), the diffracting region within a grain intersected by a pencil beam is more accurately described as a line segment passing through the grain. Consequently, the resulting diffraction spot on the detector corresponds to the integration of the diffracting signal across this line, and the task of reconstructing intra-grain orientation and strain from s3DXRD data inherently involves a tomographic challenge. Not acknowledging the tomographic nature of this problem can result in artifacts in the reconstruction of intra-grain orientation and strain (Henningsson *et al.*, 2020 ▸).

The Friedel pair method does not offer a direct resolution to this issue. Tomographic inversion methods (Henningsson *et al.*, 2020 ▸; Henningsson & Hendriks, 2021 ▸; Henningsson & Hall, 2023 ▸) have been proposed to address this problem, allowing more robust fitting of the intra-grain strain and orientation field. However, these methods might also be biased, particularly in highly deformed materials. In such cases, the assumption that the diffracting region within a grain intersected by a pencil beam forms a continuous segment can become invalid. Due to significant intra-grain misorientation, only sub-portions of the X-ray path within the grain would be positioned in the Bragg diffraction condition at a given rotation angle. Thus, the actual diffracting region at a given 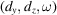 scanning position constitutes only a sub-region, possibly discontinuous, of the ray path through the grain. Developing a robust method to accurately fit the intra-grain lattice strain and orientation fields in such a case remains a challenge.

A possible direction for future improvements can nevertheless be proposed, building upon the method introduced in this article. Instead of relocating the source of paired diffraction peaks to a discrete point in the sample, one could envisage defining the peak source position as a probability density function over the 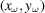 coordinates in the sample frame. This probability function should take into account the various sources of uncertainty related to detector pixel size, distance and center, as well as the spatial extent of the diffraction spot on the detector. Then, for a given point **P**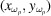 in the sample, the fitting of the phase and lattice vectors would be based on the subset of peaks associated with a non-zero probability at this position, weighting each peak contribution using the value of the probability density function at point **P**.

Put another way, this would spread the contribution of each peak over an area wider than one single pixel. The kernel smoothing method introduced in this article for local phase matching and indexing already does something similar but in a very rough way: using an 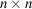 kernel is equivalent to assigning to each peak a uniform probability density over an 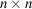 pixel region. However, this probability density is the same for each peak, regardless of diffracting intensity and peak shape on the detector. Using probability functions defined on a per-peak basis would allow a more accurate and physically consistent characterization of intra-grain lattice properties.

#### 4.2.3. Handling complex phases

Finally, another limitation of the technique pertains to phase mapping and indexing of complex phases such as feldspars, which have low symmetry and form solid solutions. As demonstrated by the phase maps (Figs. 7 ▸ and 8 ▸), dealing with these phases is challenging and the mapping strategy used resulted in poor outcomes, particularly evident in the high-resolution map (sample WG102 in Fig. 7 ▸). The poor results obtained with these phases probably stem from two issues.

First, the low symmetry of feldspars – orthoclase is monoclinic and oligoclase is triclinic – leads to a multitude of independent Bragg scattering angles corresponding to different {*hkl*}. Peak selection masks based on a simple angular threshold around each Bragg peak end up covering a substantial portion of the 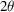 spectrum, with considerable overlaps between these two phases. A more optimized approach could involve selecting only diffraction data from the *N*-strongest Bragg peaks or adjusting the width of the 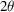 threshold for each peak on the basis of modeling of the full X-ray spectrum of each phase, to achieve a more accurate peak selection in each phase mask. Alternative decision criteria could also be used to select the best-matching phase over a given pixel. For the sake of computation efficiency, we simply took the total peak count, weighted by intensity, over each binary phase mask, retaining the phase with the highest completeness on each pixel. Other procedures could be considered, for instance retaining the phase that minimizes the sum of the 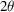 distance between each measured *hkl* reflection and the closest pre-computed Bragg peak.

Second, these minerals form solid solutions spanning a wide range of possible compositions, resulting in variations of the crystal lattice parameters. Intra-grain composition zoning or the coexistence of multiple generations of the same mineral with different compositions are extremely common in rocks. These heterogeneities imply that the lattice parameters of a given phase are not constant across the entire sample, thereby adding complexity to phase mapping. The combination of s3DXRD and X-ray fluorescence tomography (XRF–CT) within a multi-modal acquisition framework could be a promising approach to address this challenge. Mineral segmentation methods based on multi-channel X-ray fluorescence maps – obtained from electron microprobe analyzers or X-ray micro-fluorescence instruments – are relatively straightforward, assuming the minerals being analyzed are not polymorphs of the same composition (Lanari *et al.*, 2014 ▸; Lanari *et al.*, 2019 ▸; Lanari *et al.*, 2024 ▸). When combined with s3DXRD, this multi-modal X-ray mapping approach would offer a comprehensive *in situ* characterization of phase, composition, orientation and strain within rocks and other polycrystalline materials, paving the way for fully integrative 3D *in situ* mineralogy.

## 5. Conclusions

The application of synchrotron 3DXRD/HEDM techniques to complex, polyphase materials, especially natural rock samples, represents a significant challenge. We have presented here a strategy to address these complexities, based on two key principles. First, using the geometric properties of *hkl*, *h**k**l* Friedel pairs, both the precise orientation and the origin of individual scattering vectors in a s3DXRD dataset can be recovered, allowing per-pixel fitting of crystallographic properties, in a manner that is independent of grain definitions. We have introduced a new algorithm which allows efficient identification of Friedel pairs in datasets containing up to 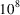 peaks, with relatively good completeness (70% to 80% of peak match). Second, overlapping 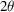 conflicts between different phases, which are particularly prominent in geological samples and hamper accurate phase segmentation, are better resolved on a per-pixel basis rather than at the whole sample scale. Using an appropriate decision criterion for each pixel, accurate phase maps can be obtained, even when large 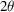 conflicts exist between different phases.

The application of the s3DXRD technique on fractured granite samples, a representative complex geological material, demonstrates the effectiveness of the method in addressing the above-mentioned challenges. Despite the inherent complexities of multi-phase materials and highly deformed structures, the s3DXRD technique offers robust phase and orientation mapping capabilities, providing valuable insights into the internal structure and behavior of such materials. However, these capabilities reach their limits when dealing with particularly complex phases such as feldspar minerals, highlighting the need for further improvement and development. While achieving complete and accurate segmentation of these phases seems within reach, accurately fitting their lattice orientation and strain remains a challenge.

## Supplementary Material

Supplementary materials. DOI: 10.1107/S1600576724009634/nb5388sup1.pdf

LTP Project BREAK@ESRF, experimental session 21-04-2023. Includes 3D X-ray diffraction data for sample WG102: https://doi.org/10.15151/ESRF-ES-1051064308

LTP Project BREAK@ESRF, experimental session 04-10-2022. Includes 3D X-ray diffraction data for sample WGSI3: https://doi.org/10.15151/ESRF-ES-902986930

## Figures and Tables

**Table 1:** Acquisition parameters for the two Westerly granite samples WGSI3 and WG102

Sample	WG102	WGSI3
Workstation	Nanofocus	3DXRD
Detector	Eiger2 X CdTe 4M	FReLoN 2K
Beam size (µm)	10	50
Beam energy (keV)	43.56	43.57
ω step size (°)	0.05	0.8
ω range (°)	[0–360]	[0–360]
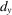 step size (µm)	10	50
*y* scanning range (mm)	6.21	5.45
Acquisition time (1 slice)	4h30	2h30

**Table 2:** Crystallographic phases in granite samples WGSI3 and WG102

Phase	Space group	*a* (Å)	*b* (Å)	*c* (Å)	α (°)	β (°)	γ (°)
Quartz	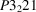	4.913	4.913	5.412	90	90	120
Oligoclase	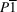	8.154	12.823	7.139	94.06	116.50	88.59
Orthoclase	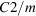	8.589	13.013	7.197	90	116.02	90
Biotite	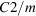	5.355	9.251	10.246	90	100.15	90
Magnetite	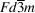	8.396	8.396	8.396	90	90	90
Aluminium	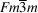	4.050	4.050	4.050	90	90	90

## Data Availability

The Python module presented in this article is available at https://github.com/jbjacob94/pf_3dxrd.git. The original syn­chrotron data and metadata for samples WGSI3 and WG102 are available at https://doi.org/10.15151/ESRF-ES-1051064308 and https://doi.org/10.15151/ESRF-ES-902986930.
