# Supplementary material for: Exploiting Friedel pairs to interpret scanning 3DXRD data from complex geological materials
Source: J Appl Crystallogr. 2024 Nov 8;57(Pt 6):1823–40. doi: 10.1107/S1600576724009634 (PMC11611280; doi:10.1107/S1600576724009634)

JOURNAL OF
APPLIED
CRYSTALLOGRAPHY

Volume 57 (2024)

Supporting information for article:

Exploiting Friedel pairs to interpret scanning 3DXRD data from complex geological materials

Jean-Baptiste Jacob, Jonathan Wright, Benoît Cordonnier and François Renard

Supplementary Materials

1 - Detailed Flowchart

The flowchart below provides a more comprehensive overview of the processing workflow compared to the simplified sketch in Fig. 1. Raw 2D diffraction images, stored in an HDF5 file, undergo the five steps outlined in Fig. 1: peak segmentation, Friedel pairs matching, pixel-by-pixel phase labeling, pixel-by-pixel UBI fitting (local indexing of unit cell matrices), and grain mapping. At each stage, new files are generated, or additional data are appended to existing files, as detailed in the white boxes. Phase mapping and local indexing require a list of pre-determined crystal structures, typically provided as CIF files.

In cases where the material's crystal structures are unknown, azimuthal integration can be used to generate a 1D X-ray diffraction spectrum. This spectrum can then be compared to a crystal structure database to identify the phases and refine the average cell parameters of each phase before indexing. This step can be performed either on the raw diffraction images using pyFAI, or using the distribution of diffraction peaks obtained after segmentation. For large samples with significant peak offsets on the detector due to the parallax effect, it is preferable to chose the second option, using offset-corrected peak positions obtained after identification of Friedel pairs.

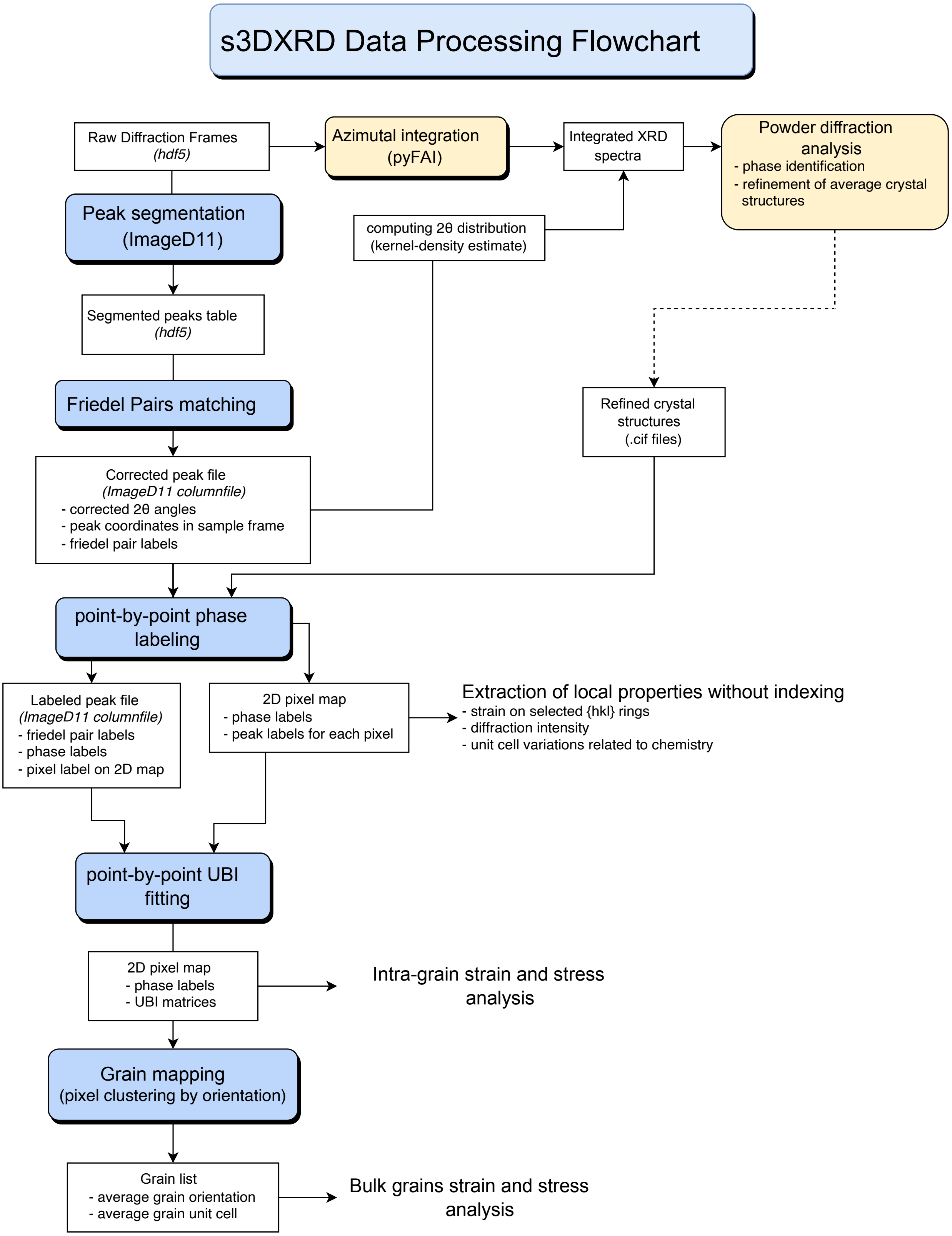
s3DXRD Data Processing Flowchart
Raw Diffraction Frames
(hdf5)
Azimutal integration
(pyFAI)
Integrated XRD spectra
Powder diffraction analysis
- phase identification
- refinement of average crystal structures
Peak segmentation
(ImageD11)
computing 2θ distribution
(kernel-density estimate)
Segmented peaks table
(hdf5)
Friedel Pairs matching
Refined crystal structures
(.cif files)
Corrected peak file
(ImageD11 columnfile)
- corrected 2θ angles
- peak coordinates in sample frame
- friedel pair labels
point-by-point phase labeling
Labeled peak file
(ImageD11 columnfile)
- friedel pair labels
- phase labels
- pixel label on 2D map
2D pixel map
- phase labels
- peak labels for each pixel
Extraction of local properties without indexing
- strain on selected {hkl} rings
- diffraction intensity
- unit cell variations related to chemistry
point-by-point UBI fitting
2D pixel map
- phase labels
- UBI matrices
Intra-grain strain and stress analysis
Grain mapping
(pixel clustering by orientation)
Grain list
- average grain orientation
- average grain unit cell
Bulk grains strain and stress analysis

2. Raw diffraction spots

The processing workflow described above operates with discrete peak positions, corresponding to the center of mass of actual diffraction spots. This approach is an approximation, as real diffraction spots occupy a non-zero area on the detector, exhibiting azimuthal spread due to orientation variations within the diffracting volume and radial spread due to lattice strain. If the diffraction spots shapes deviate significantly from sharp circular blobs, this discrete peak approximation may lead to inaccurate results.

Below is a representative sample of raw diffraction peaks for the two samples discussed in the paper (WGSI3: Westerly Granite; WG102: Westerly Granite in an aluminum gasket). Most of the peaks from the granite appear relatively sharp and show aspect ratios close to 1. However, the peaks from the aluminum gasket in sample WG102 are highly elongated in the azimuthal direction, which likely explains the inconsistent indexing reported in the study.

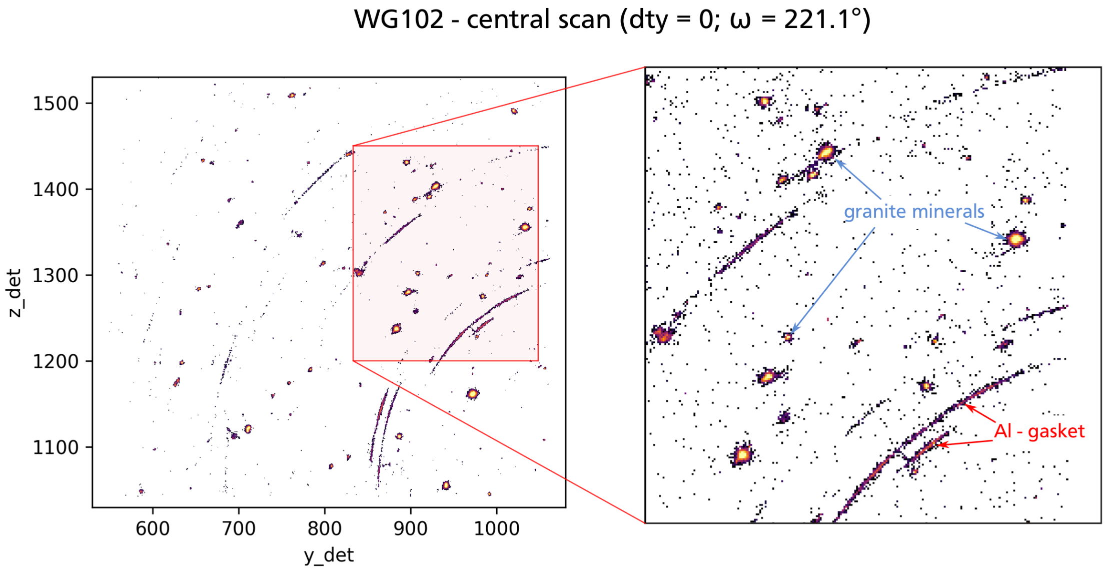

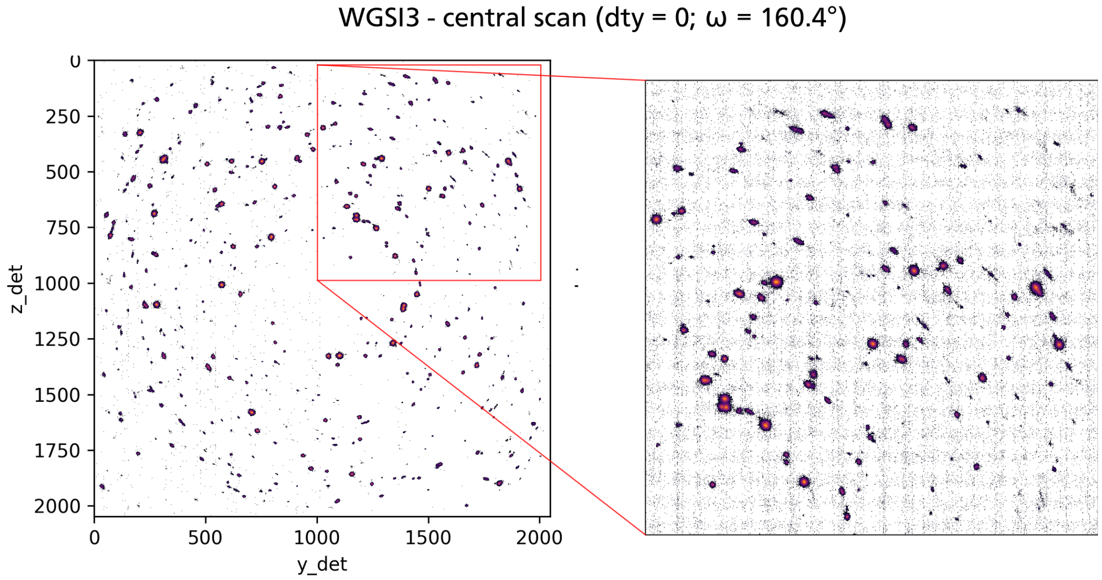

3. local g-vectors

Assigning diffraction peaks to specific pixel positions within the sample enables peak selection on a per-pixel basis. An example is shown below for a 3x3 pixel domain (red) within a quartz grain (blue) in sample WGSI3. The diffraction vectors (g-vectors) selected from this domain are plotted in 3D reciprocal space, as well as on a stereographic projection onto the gx-gy plane, illustrating that the data is consistent with diffraction from a single crystal. In these plots, each spot corresponds to a specific (hkl) Miller index of the quartz lattice.

Each spot contains numerous diffraction peaks, highlighting the high redundancy of the diffraction data obtained through s3DXRD. This redundancy means that each (hkl) plane is sampled multiple times, enhancing the robustness of local indexing. The level of redundancy increases with the size of the kernel around the central pixel. However, as the selection volume increases, so does the orientation spread of the lattice within that volume, particularly in the presence of significant orientation gradients. This can lead to greater uncertainty in the orientation and magnitude of the fitted lattice vectors.

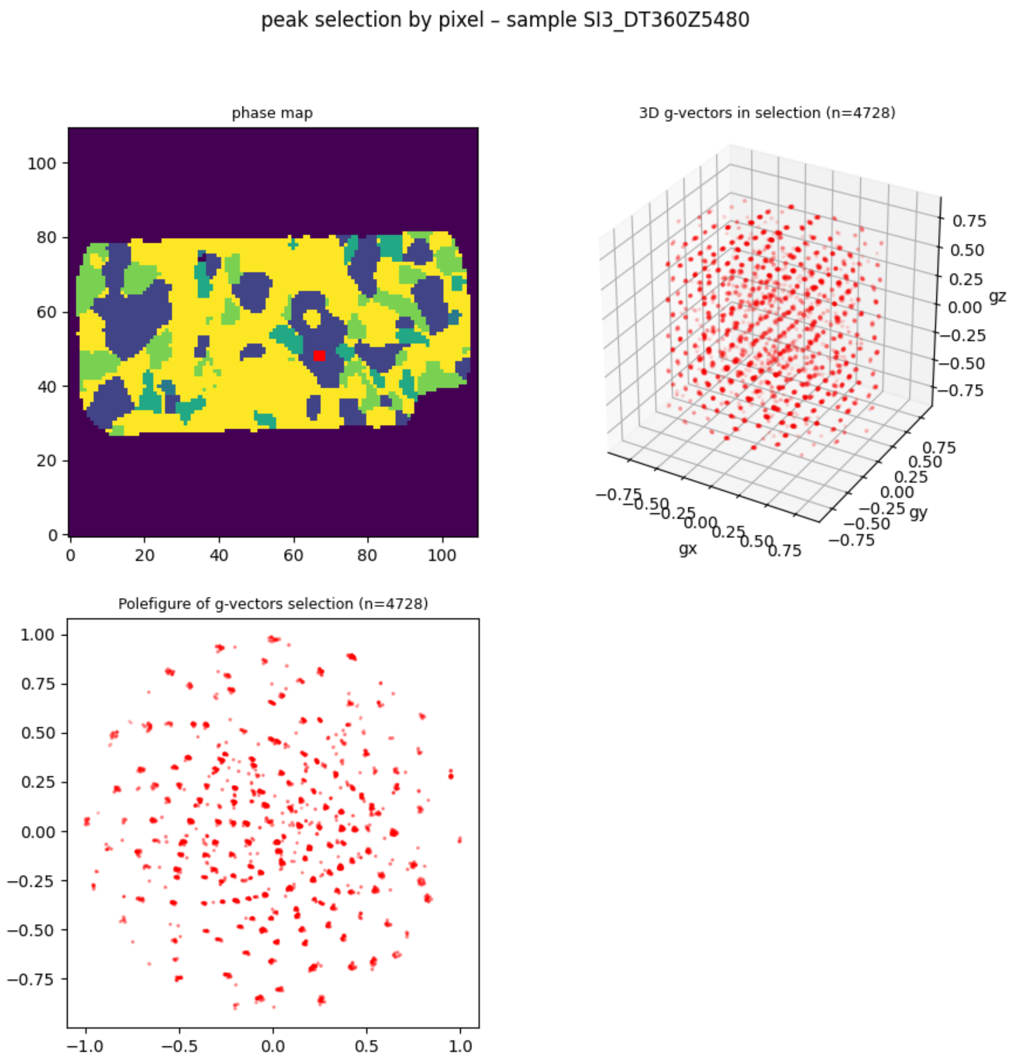

Supplement: Supplementary file 1 [file j-57-01823-sup1.pdf]
